# Marine Natural Products as Potent Anticancer Agents (2020–2024): Structural Diversity, SARs and Target Prediction

**DOI:** 10.3390/md24050173

**Published:** 2026-05-10

**Authors:** Zimeng Huang, Yijing Du, Junzhe Hu, Leyi Ying, Binying Zhou, Yi Hua, Hong Wang, Zhikun Yang

**Affiliations:** 1 College of Pharmaceutical Science & Jianxing Honors College, Zhejiang University of Technology, Hangzhou 310014, China; 202200530509@zjut.edu.cn (Z.H.); 302023514030@zjut.edu.cn (Y.D.); 302023514167@zjut.edu.cn (J.H.); yinglele2000@163.com (L.Y.); 302024514017@zjut.edu.cn (B.Z.); huayi@zjut.edu.cn (Y.H.); 2 Zhejiang-Egypt Joint Laboratory on Intelligent Discovery of Marine Drugs, Hangzhou 310014, China; 3 Zhejiang Key Laboratory of Green Manufacturing Technology for Chemical Drugs, Hangzhou 310014, China

**Keywords:** marine natural product, structural elucidation, anticancer activity, structure–activity relationship, skeleton analysis, target prediction

## Abstract

In recent years, Marine Natural Products (MNPs) have emerged as a significant source for anticancer drug discovery, as many natural products can offer structural diversity, unique mechanisms of action, and relatively low toxicity. This article provides a systematic review of MNPs with reported anticancer activities from 2020 to 2024. These compounds are classified into seven major categories: terpenoids, alkaloids, sterols, polyketides, peptides and proteins, polysaccharides, and macrolides. For each category, we elaborate on the marine sources, structural identification, in vitro anticancer activity, and preliminary structure–activity relationships. We found that sponges and marine-derived fungi are the most abundant sources of highly active compounds. Furthermore, knowledge graph-based analysis reveals that oxygen- and nitrogen-containing heterocycles constitute the core pharmacophores, and target prediction further indicates that MNPs exert anticancer effects through coordinated modulation of a multi-target network involving kinases, proteasomes, and nuclear receptors. This review contributes significantly to a deeper understanding of recent advances (2020–2024) in MNPs and provides critical guidance for promoting the development of innovative anticancer drugs derived from marine resources.

## 1. Introduction

Cancer remains one of the most formidable public health challenges worldwide. Common types such as breast, lung, and colorectal cancer are not only prevalent but also persistently threaten human health and quality of life. The occurrence and progression of cancer result from the interplay of multiple cellular and molecular mechanisms. Processes including inhibition of kinases, DNA damage, apoptosis, cell cycle arrest, and ROS generation collectively constitute the core biological basis of tumor initiation and progression [1]. According to statistics, approximately 20 million new cancer cases and 9.7 million cancer-related deaths were reported globally in 2022. With the intensification of population aging, lifestyle changes, and persistent environmental risk factors, the cancer burden is projected to continue rising in the coming decades, posing ongoing pressure on global public health systems [2]. Although advances have been made in surgery, chemotherapy, radiotherapy, and targeted therapy, current treatments still face significant challenges such as insufficient efficacy, strong toxicity, and the development of drug resistance, which severely impact patient quality of life [3,4]. Therefore, there is an urgent need to discover novel anticancer agents that are highly effective, low in toxicity, and with innovative mechanisms of action.

**Figure 1:**
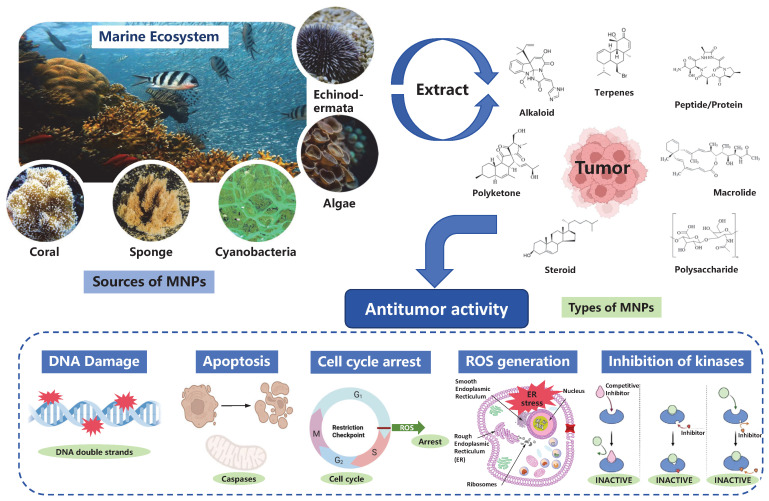
Marine natural products as potent anticancer agents.

Against this backdrop, natural products have emerged as an important source for anticancer drug discovery, largely because certain classes of them are known for structural diversity, unique mechanisms of action, and relatively low toxicity [5]. Historically, over 60% of anticancer drugs are derived directly or indirectly from natural products [6]. However, the exploration of terrestrial resources has approached saturation, leading researchers to turn their attention to the oceans, which cover more than 70% of the Earth’s surface and harbor extremely rich biodiversity [7,8]. The unique marine environment, characterized by high salinity, high pressure, and low oxygen, drives marine organisms to evolve and produce a vast array of structurally novel and biologically potent secondary metabolites [9]. These marine natural products (MNPs) often exhibit structural features rarely found in terrestrial counterparts, such as longer carbon chains, complex ring systems, and abundant halogen atoms, endowing them with distinctive pharmacological activities and great potential as lead compounds (Figure 1) [9,10].

The marine ecosystem harbors a wealth of biological resources, including corals, sponges, cyanobacteria, algae, and various invertebrates, all of which serve as valuable sources of bioactive MNPs [11]. Numerous compounds with antiviral, antifungal, and anticancer activities have been discovered from these marine resources. Since the approval of the first marine-derived anticancer drug Cytarabine in 1969, a total of 11 marine-derived anticancer drugs have been successfully applied in clinical practice [12,13]. These drugs encompass a variety of mechanisms of action, which can be broadly categorized into four main classes: The first class consists of nucleoside analogs developed based on the structural foundation of nucleosides derived from marine sponges, including Cytarabine, Vidarabine, Fludarabine, and Nelarabine, which are primarily used to treat various types of leukemia [14,15,16]. The second class is the microtubule inhibitor *Eribulin* mesylate, derived from Halichondrin B extracted from the sponge Halichondria okadai, used for the treatment of metastatic breast cancer and liposarcoma [13,17]. The third class includes DNA alkylating agents originating from the tunicate Ecteinascidia turbinata, namely Trabectedin and its synthetic analog Lurbinectedin, used for the treatment of soft tissue sarcoma, ovarian cancer, and metastatic small cell lung cancer, respectively [18,19]. A particularly successful class is that of antibody-drug conjugates (ADCs), whose cytotoxic payloads are microtubule-disrupting agents (Monomethyl Auristatin E/F) derived from the marine mollusk Dolabella auricularia. This class of drugs achieves targeted delivery through antibodies and includes Brentuximab vedotin, Polatuzumab vedotin, Enfortumab vedotin, and Belantamab mafodotin, which are used to treat various malignancies such as Hodgkin’s lymphoma, diffuse large B-cell lymphoma, advanced urothelial carcinoma, and relapsed multiple myeloma [20,21,22]. The successful marketing of these drugs fully demonstrates the great potential and structural diversity of MNPs in anticancer drug research and development.

Currently, numerous studies focus on novel marine-derived anticancer compounds, including reviews on marine-derived bisindole alkaloids [23], marine phenolic compounds and their derivatives [24], indole alkaloids [25], compounds derived from brown algae [26], and compounds isolated from sponges [27]. However, a systematic review of MNPs with anticancer activities reported between 2020 and 2024 is lacking. Therefore, systematically reviewing recently discovered marine anticancer natural products and analyzing their chemical characteristics and potential mechanisms of action using modern computational tools is of great significance for accelerating the drug discovery process. Based on this, this article aims to systematically review MNPs with anticancer activities discovered between 2020 and 2024. We classify these compounds into seven major structural categories: terpenoids, alkaloids, sterols, polyketides, peptides and proteins, polysaccharides, and macrolides [28,29,30,31,32,33]. For each category, we elaborate on the marine source, structural identification, in vitro anticancer activity data, and preliminary structure–activity relationships (SARs). Furthermore, we employ the Neo4j knowledge graph to perform statistical analysis on the chemical scaffolds of representative molecules and utilize the SwissTargetPrediction platform to predict their potential molecular targets. This work is intended to provide a systematic knowledge base and novel research perspectives for future anticancer drug discovery based on marine resources.

## 2. Anticancer MNPs

### 2.1. Marine-Derived Terpenoids

Marine-derived terpenoids are characterized by isoprene units as basic skeleton, and structurally cover multiple levels including monoterpenes, sesquiterpenes, diterpenes, triterpenes and higher terpenoids. Terpenoids are widely distributed in the metabolic systems of sponges, algae, fungi and their symbiotic microorganisms, presenting complex oxidation states, cyclization patterns and stereoconfiguration diversity. The unique marine environment drives their chemical evolution toward highly oxidized and structurally novel scaffolds, establishing terpenoids as a prolific source of anticancer drug leads [34].

**Figure 2:**
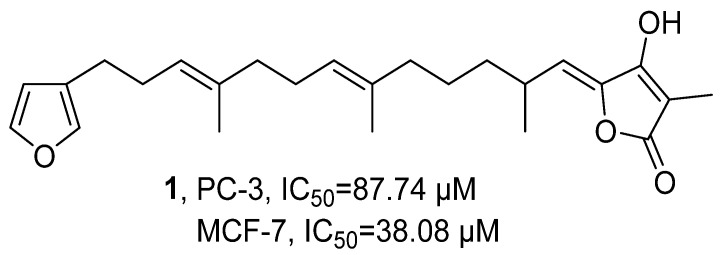
Structure of marine-derived terpenoid Variabilin (**1**).

In 2020, Beukes’ research group isolated the terpene component Variabilin (**1**) from the South African sponge *Ircinia* sp. through silica gel column chromatography and HPLC purification (Figure 2) [35]. **1** was determined to be C_25_H_34_O_4_ using HRMS, and its structure was confirmed by ^1^H NMR and ^2^D NMR. Using the WST-1 method, the activity assessment showed that **1** had a significant inhibitory effect on prostate cancer cells PC-3, with an IC_50_ value of 87.74 µM, but its efficacy was inferior to Ceramide which refers to a class of waxy lipid compounds formed by the combination of sphingosine and fatty acids through amide bonds (IC_50_ = 4.81 µM); moreover, **1** also exhibited significant inhibitory activity against breast cancer cells MCF-7 (IC_50_ = 38.08 µM), which was comparable to the standard compound Ceramide (IC_50_ = 33.61 µM).

**Figure 3:**
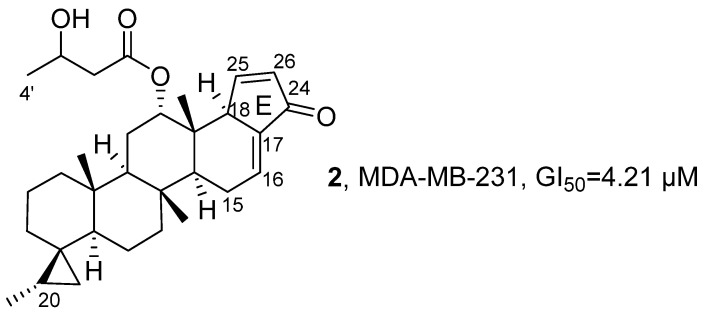
Structure of marine-derived terpenoid **2**.

In 2021, Lee’s research group isolated 15 new scalarane-type sesterterpenoids from the marine sponge *Dysidea* sp. collected from Bohol Island using methanol and dichloromethane extracts (Figure 3) [36]. Taking **2** as a reference, its molecular formula was determined to be C_31_H_44_O_4_ using HRMS, and the core cyclopentenone E-ring skeleton was inferred based on the correlation signals in the HMBC spectrum between H-25 and C-17/C-18/C-24, and between H-26 and C-17/C-24/C-25, which was further confirmed by the H-18-H-25-H-26 coupling chain observed in the ^1^H−^1^H COSY spectrum. Using CCK-8 assay, the activity assessment showed that, compared with other compounds, **2** exhibited the strongest cytotoxic activity against MDA-MB-231 cells, with a GI_50_ value of 4.21 μM. SAR studies indicated that the Δ^15,16^ double bond generally reduced activity, the methylene elongation at the C-4′ position helped enhance activity. However, SAR analysis also suggested that oxidation at C-20 reduced cytotoxic potency relative to the non-oxidized analogs, indicating that the hydrophobic substitution pattern at this position is important for activity.

**Figure 4:**
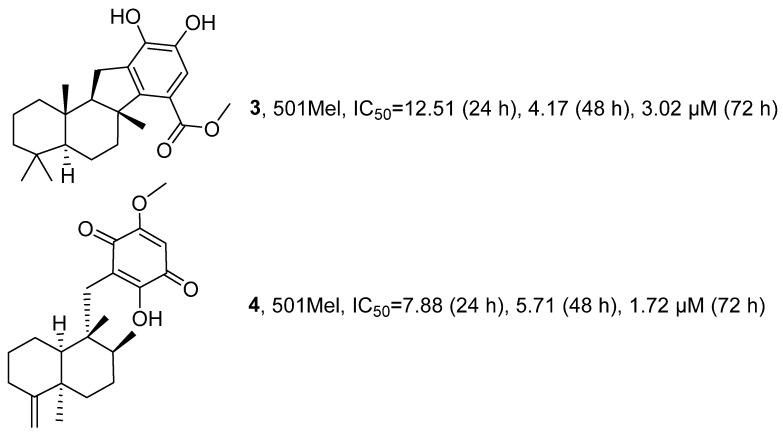
Structures of marine-derived terpenoids Pelorol (**3**) and 5-epi-Ilimaquinone (**4**).

In 2022, Carpi’s research group conducted in-depth investigations on two existing terpenoids, Pelorol (PEL) (**3**) and 5-epi-Ilimaquinone (EPI) (**4**), isolated from the methanol exudates of the marine sponge *Dactylospongia elegans*, building upon previous explorations (Figure 4) [37]. Activity evaluation results showed that when tested on human 501 Mel melanoma cells, the average IC_50_ values for **3** after 24, 48, and 72 h of administration were 12.51 µM, 4.17 µM and 3.02 µM, respectively, whereas the average IC_50_ values for **4** during the same time periods were 7.88 µM, 5.71 µM and 1.72 µM, respectively.

**Figure 5:**
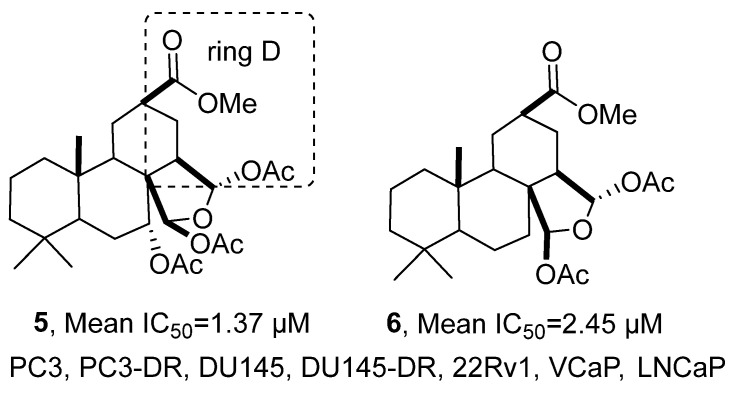
Structures of marine-derived terpenoids **5** and **6**.

In 2022, Dyshlovoy’s research group isolated six classes of spongian-type diterpenoids from the methanol extract of the marine sponge *Spongionella* sp., including five known compounds (Figure 5) [38]. The molecular formula of the new compound **5** was determined by HRMS to be C_27_H_40_O_9_, and ^1^H NMR spectrum revealed the presence of three sets of methyl groups, three sets of acetyl methyl groups, one methoxycarbonyl group, and two sets of acetal protons. The HMBC spectrum confirmed the attachment of an additional acetoxy group to the C-7 position, and further information from HSQC, COSY, and NOESY spectra was integrated to deduce its relative configuration and the structural feature of the opened D-ring. Cell viability was examined by MTT assay. Compared with the positive control drug cisplatin (average IC_50_ = 8.40 μM), compounds **5** and **6** exhibited stronger cytotoxic activity against multiple prostate cancer cell lines (PC3, PC3-DR, DU145, DU145-DR, 22Rv1, VCaP, LNCaP), with average IC_50_ values of 1.37 μM and 2.45 μM, respectively. SAR studies indicated that the opening of the D-ring modification plays a crucial role in enhancing the cytotoxicity and selectivity of these compounds.

**Figure 6:**
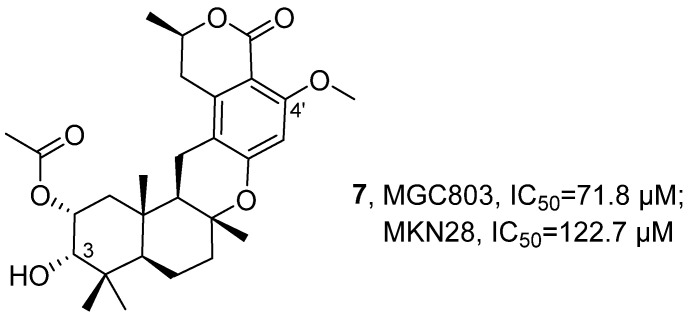
Structure of marine-derived terpenoid Taladrimanin A (**7**).

In 2022, Wang’s research group isolated a novel methylenediterpene compound, Taladrimanin A (**7**), and 11 related known compounds from the marine-derived fungus *Talaromyces* sp. HM6-1-1 (Figure 6) [39]. For **7**, its structure was determined by various spectroscopic techniques: HRMS indicated its molecular formula as C_28_H_38_O_7_ with an unsaturation degree of 10; NMR (including ^1^H NMR, ^13^C NMR, DEPT, and HSQC) revealed that it contained 7 methyl groups, 6 methylene groups, and 10 non-protonated carbons; COSY correlation signals identified four independent spin systems; HMBC spectra further revealed a drimane-type sesquiterpene unit, which was connected to an ester carbonyl and two oxygenated sp^2^ hybridized quaternary carbons. Combining the sesquiterpene and isochromenone skeletons, as well as the substitution at C-3 and C-4′ positions, **7** was ultimately determined to be a hybrid compound of a pentaketide-peptide and a terpene. Bioactivity was examined by CCK8 assay. Compared with the positive control drug cisplatin (IC_50_ = 6.2 μΜ, 19.7 μΜ), compound 7 exhibited weaker anti-tumor activity against gastric cancer cells MGC803 and MKN28, with IC_50_ values of 71.8 μM and 122.7 μM, respectively. SAR analysis indicated that the activity of **7** originated from the hybridization of the unique drimane-type sesquiterpene unit and an isochromenone unit derived from polyketides.

**Figure 7:**
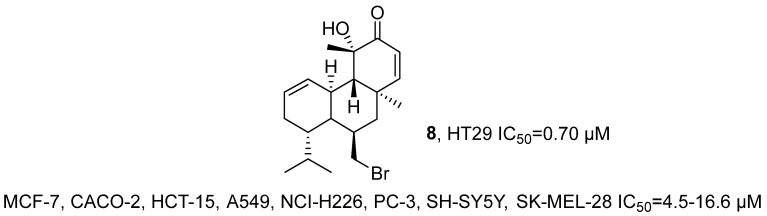
Structure of marine-derived terpenoid sphaerococcenol A (**8**).

In 2022, Pedrosa’s research group isolated the brominated diterpene compound sphaerococcenol A (**8**) from the red alga *Sphaerococcus coronopifolius* Stackhouse 1797 (Figure 7) [40]. After extraction, the structure of the compound was characterized by MS and NMR techniques and confirmed by comparison with the reports by Rodrigues [41]. The results of the activity evaluation indicated that **8** exhibited broad-spectrum cytotoxic activity against 8 tumor cell lines (MCF-7, CACO-2, HCT-15, A549, NCI-H226, PC-3, SH-SY5Y, SK-MEL-28) with IC_50_ values ranging from 4.5 to 16.6 μM. Notably, it demonstrated significant anti-tumor activity against colon cancer stem cell HT29 spheres with an IC_50_ of 0.70 μM.

**Figure 8:**
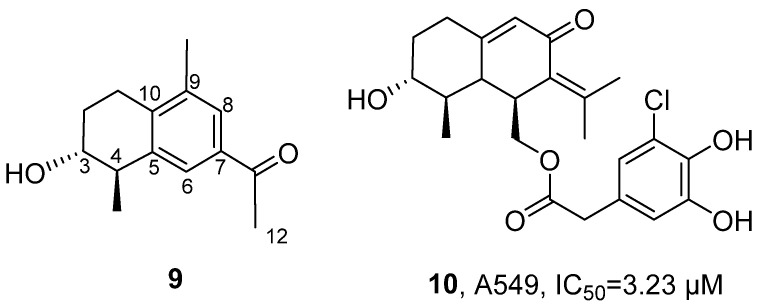
Structures of marine-derived terpenoids Copteremophilane A (**9**) and Copteremophilane H (**10**).

In 2022, Lin’s research group isolated 12 new compounds from the ethyl acetate extract of the yellow-green *Penicillium copticola* fungus that lives symbiotically with marine sponges (Figure 8) [42]. Among them, 10 compounds were identified as furostanol sesquiterpenes, and the other two compounds were confirmed as glucosides. For Copteremophilane A (**9**), molecular formula was determined as C_14_H_18_O_2_ by HRMS; ^13^C NMR and DEPT spectra showed 14 carbon signals, including six aromatic carbons, one ketone carbonyl carbon, and seven alkyl carbons. Through two meta coupling aromatic proton signals (H-6 and H-8), a tetrasubstituted aromatic ring was identified; further combined with HMBC and COSY correlation signals, the presence of a methyl group at C-9, acetoxy group at C-7, methyl group at C-4, and hydroxyl group at C-3 was determined, and it was inferred that the C-5 and C-10 units fused with the aromatic ring at the C-5 and C-10 positions to form a cyclohexene ring; NOE correlation signals indicated that H_3_-15 was oriented towards OH-3. Comparison of the experimental ECD data to those calculated for (3*R*, 4*R*, 5*S*)-8 established 3*R*, 4*R* and 5*S* configurations for **9**. The activity evaluation was examined by MTT assay. Results showed that Copteremophilane H (**10**) had selective inhibitory activity against A549 cells, with an IC_50_ value of 3.23 μM. The SAR analysis indicated that the introduction of the benzene acetic acid unit significantly enhanced the anti-tumor effect of furostanol compounds, and the position of substitution and the composition of the acyl group had a significant impact on their selective inhibitory activity.

**Figure 9:**
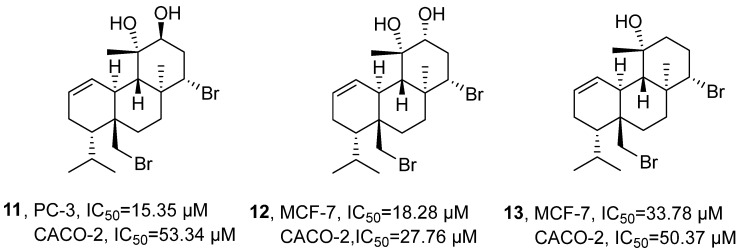
Structures of marine-derived terpenoids 12*S*-hydroxy-bromosphaerol (**11**), 12*R*-hydroxy-bromosphaerol (**12**), and bromosphaerol (**13**).

In 2022, Pedrosa’s research group isolated three brominated diterpenoids (12*S*-hydroxy-bromosphaerol, 12*R*-hydroxy-bromosphaerol, and bromosphaerol) (**11–13**) from the dichloromethane extract of *Sphaerococcus coronopifolius* (Figure 9) [43]. Through the comprehensive application of NMR (^1^H and ^13^C NMR, COSY, HMBC, and HSQC) techniques, their chemical structures were finally determined. Activity evaluation results indicated that **12** exhibited significant anti-tumor activity, with an IC_50_ of 18.28 µM in MCF-7 cells and 27.76 µM in Caco-2 cells. For **11**, the IC_50_ was 15.35 µM in PC-3 cells and 53.34 µM in Caco-2 cells. In comparison, **13** showed IC_50_ values of 33.78 µM in MCF-7 cells and 50.37 µM in Caco-2 cells.

**Figure 10:**
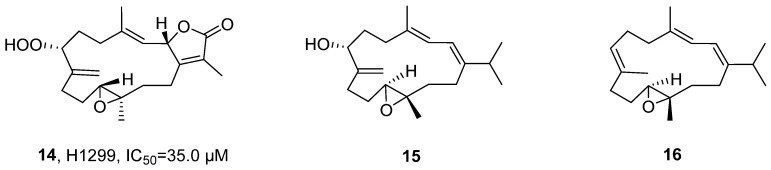
Structures of marine-derived terpenoids Sarcomililatin B (**14**), Sarcoboettgerol D (**15**) and Sarcoboettgerol E (**16**).

In 2023, Wang’s research group isolated two undescribed cembranoids, Sarcoboettgerol D (**15**) and Sarcoboettgerol E (**16**), along with four known related analogs, from the acetone extract of *Sarcophyton boettgeri* (Figure 10) [44]. The structural elucidation, exemplified by **15**, began with the determination of its molecular formula as C_20_H_32_O_2_ by HRMS. The characteristic functional groups, including a conjugated diene and an exocyclic double bond, as well as the planar structure were established through comprehensive NMR analysis (^1^H, ^13^C, COSY, and HMBC). Subsequently, the relative and absolute configurations were unambiguously assigned using combined QM-NMR and TDDFT-ECD calculations. In vitro activity evaluation indicated that Sarcomililatin B (**14**) exhibited weak cytotoxicity against H1299 lung cancer cells, with an IC_50_ value of 35.0 μM.

In terpenoids, hydroxyl, ketone, double bond, and ester groups are the most frequent motifs, forming a highly oxidized core. The most active terpenoids originate mainly from sponges (e.g., Ircinia, Dysidea, Spongionella) and red alga (Sphaerococcus coronopifolius). D-ring opening and bromination greatly enhance selectivity and cytotoxicity. Trends from 2020 to 2024 include that detailed SAR studies (e.g., Δ^15,16^ double bond reduces activity, C-4′ methylene elongation increases activity), absolute configuration determination by QM-NMR and ECD calculations.

### 2.2. Marine-Derived Alkaloids

Marine-derived alkaloids are a class of natural products with nitrogen-containing heterocycles as the basic framework. Their structural types are diverse, including indoles, quinolines, quinazolines, and polycyclic amines, etc. Marine-derived alkaloids often exhibit complex characteristics such as halogen substitution, high oxidation states, and multi-center chirality, which give them remarkable marine chemical uniqueness. They are mainly discovered from sponges and their symbiotic microbial systems and are an important source of anticancer drug lead compounds.

**Figure 11:**
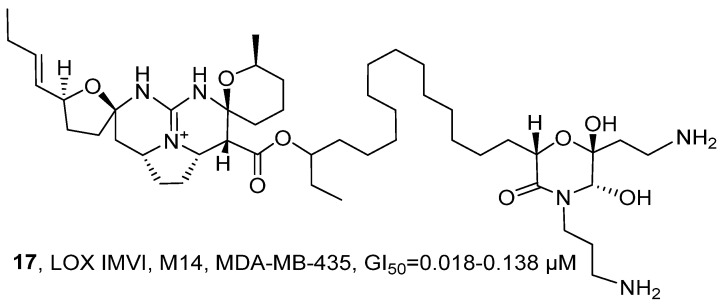
Structure of marine-derived alkaloid Monanchocidin A (**17**).

In 2020, T. Hamann’s research group isolated a biogenic alkaloid named Monanchocidin A (**17**) from the ethanol extract of the *Monanchora* genus sponges in the subarctic waters (Figure 11) [45]. The structure of **17** was determined by comparing it with its known standard data set (including ^1^H NMR, ^13^C NMR and HRMS). Compared with the positive control drug paclitaxel, **17** exhibited stronger cytotoxic activity against LOX IMVI, M14, and MDA-MB-435 cells, with GI_50_ values ranging from 0.018 to 0.138 µM. By comparing the SAR with the structurally similar guanidine-based alkaloid Ptilomycalin A, it was found that they had a high Pearson correlation (0.638), indicating that the pentacyclic guanidine core skeleton and the spermine fragment at the end are crucial for its anti-tumor activity.

**Figure 12:**
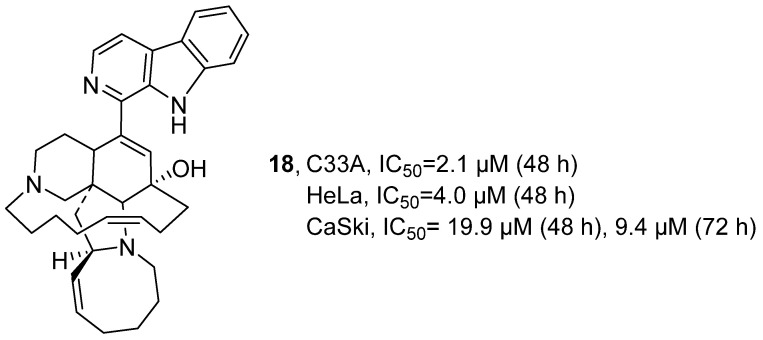
Structure of marine-derived alkaloid Manzamine A (**18**).

In 2020, T. Hamann’s research group isolated Manzamine A (**18**) from the Indonesian sponge *Acanthostrongylophora* (Figure 12) [46]. **18** belongs to the *β*-cycline alkaloids with complex polycyclic structures. Through techniques such as HRMS, LCMS/MS and NMR, the main components and secondary components of **18** were identified. Biological activity was evaluated by CellTiter-Glo cell viability assay. **18** demonstrated potent inhibitory activity against C33A and HeLa cell lines, with respective 48 h IC_50_ values of 2.1 μM and 4.0 μM. Against CaSki cells, its inhibitory effect was further characterized by IC_50_ values of 19.9 μM at 48 h and 9.4 μM at 72 h.

**Figure 13:**
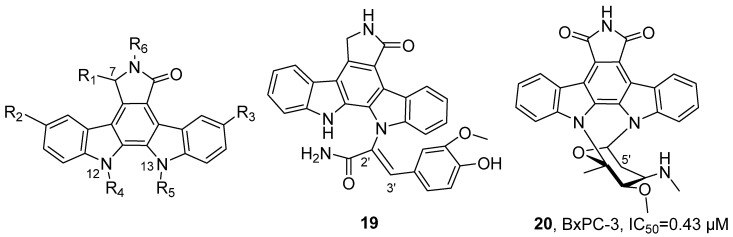
Structures of marine-derived alkaloids **19** and **20**.

In 2020, Chen’s research group isolated and identified 69 natural indolocarbazole alkaloids from the ethyl acetate extracts of four marine-derived *Streptomyces* strains, including 15 novel compounds (Figure 13) [47]. For **19**, molecular formula was determined as C_30_H_22_N_4_O_4_ with 22 degrees of unsaturation by HRMS. Further combined with NMR data, correlation spectroscopy (COSY, HMBC), and single-crystal X-ray diffraction analysis, the connectivity of its fragment structures was confirmed, including the geometry of the C2′-C3′ double bond. Activity evaluation results showed that **20** exhibited inhibitory activity against nine human tumor cell lines, with the highest sensitivity observed in pancreatic cancer cells BxPC-3 (IC_50_ = 0.43 μM). In mouse xenograft model experiments, **19** significantly inhibited tumor growth (tumor growth inhibition rate TGI = 49.6%). SAR analysis revealed that the presence of an aglycone is a key factor for maintaining activity; compounds with a disaccharide linkage at N-12 and N-13 and no substitution at the C-5′ position demonstrated stronger activity; the configuration at the C-7 position influences activity; for example, the (*S*)-methoxy substituted compound showed lower activity than its epimer; additionally, the chair conformation exhibited higher activity compared to the boat conformation.

**Figure 14:**
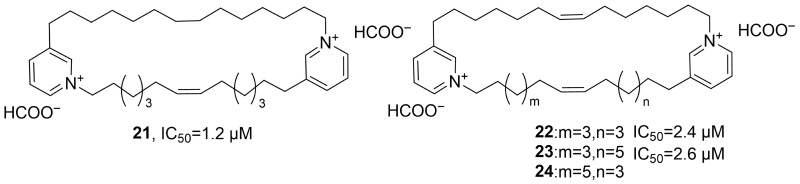
Structures of marine-derived alkaloids neopetrosidines A–D (**21**–**24**).

In 2021, Tsukamoto’s research group isolated four new pyridine alkaloids, neopetrosidines A–D (**21**–**24**), from the ethanol extract of the marine sponge *Neopetrosia chaliniformis* (Figure 14) [48]. Taking the structure analysis of neopetrosidine A as an example, HRMS determined its molecular formula to be C_38_H_62_N_2_; the ^1^H NMR spectrum showed aromatic proton signals and alkene proton signals; through COSY, HMBC and HSQC spectroscopic analysis, it was confirmed that the structure contained two 1,3-disubstituted pyridine units; combined with the ^13^C NMR chemical shift information, the double bond configuration was further determined to be the Z form. Using MTT assay, activity studies showed that **21**–**24** could significantly inhibit the growth of HeLa/Fucci2 cells, with IC_50_ values of 1.2, 2.4 and 2.6 μM, respectively.

**Figure 15:**
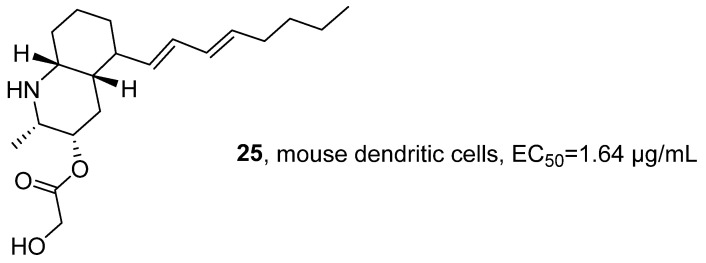
Structure of marine-derived alkaloid Lepadin A (**25**).

In 2021, Fontana’s research group isolated the marine alkaloid Lepadin A (**25**) from the methanol extract of *Clavelina lepadiformis* sp. B collected from the Tyrrhenian Sea (Figure 15) [49]. In terms of structural identification, the molecular formula was determined to be C_20_H_34_NO_3_ by HRMS, and the ^1^H NMR spectral signals confirmed the presence of the butadienyl system and two methyl groups. The activity evaluation results showed that **25** had cytotoxic effects on various cancer cell lines (such as lung cancer cells CALU-1, CALU-3, HCC827, melanoma cells MALME-3M, A375, A2058, and multiple myeloma cells KMS-12, RPMI 8226, and JJN-3); the test on D1 cells confirmed its immunomodulatory activity, with an EC_50_ of 1.64 μg/mL, while its cytotoxic activity was 4.20 μg/mL in the same cell line.

**Figure 16:**
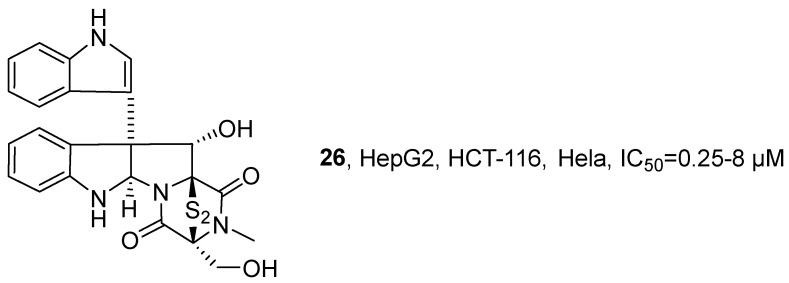
Structure of marine-derived alkaloid GQQ-792 (**26**).

In 2021, Li’s research group isolated epipolythiodiketopiperazines (ETPs) compounds from the MNP, *Tilachidium* sp., an endophytic fungus found in mangroves. GQQ-792 (**26**), identified from the MNPs, also belongs to the ETPs class of alkaloids (Figure 16) [50]. The activity evaluation results showed that **26** exhibited IC_50_ values for HepG2, HCT-116, and Hela cell lines within the range of 0.25–8 μM. The SAR study indicated that the disulfide group is the key pharmacophore, and the absence of this group would significantly reduce the inhibitory activity.

**Figure 17:**
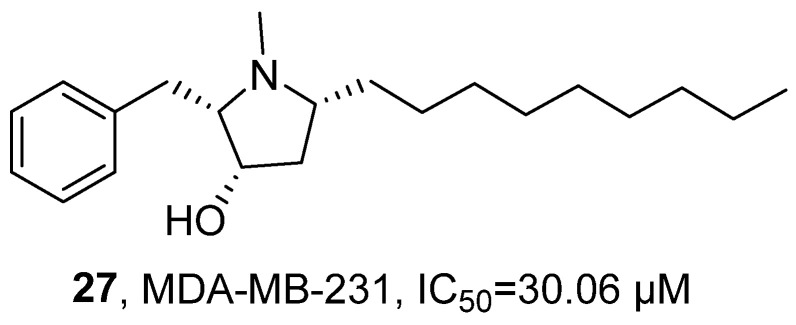
Structure of marine-derived alkaloid Preussin (**27**).

In 2023, Rocha’s research group isolated the alkaloid Preussin (**27**) from the sponge-associated fungus *Aspergillus candidus* KUFA 0062 (Figure 17) [51]. Based on previous studies [52,53,54,55,56,57,58], they further systematically evaluated its anti-tumor activity. The activity test results showed that **27** exhibited anti-tumor activity against MDA-MB-231 cells, with an IC_50_ value of 30.06 μM.

**Figure 18:**
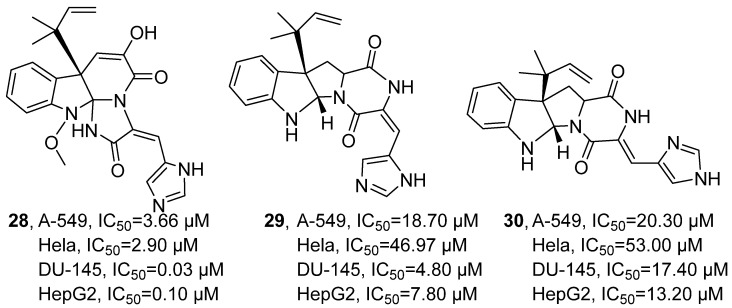
Structures of marine-derived alkaloids meleagrin (**28**), roquefortine C (ROC, **29**), and isoroquefortine C (**30**).

In 2023, A. Bogari’s research group isolated three indole-type alkaloids from the secondary metabolites of the marine fungus *Penicillium chrysogenum* S003 sourced from the Red Sea using silica gel column chromatography: meleagrin (MEL, **28**), roquefortine C (ROC, **29**), and isoroquefortine C (ISO, **30**) (Figure 18) [59]. Taking **28** as an example for compound structure confirmation, ^1^H-NMR and ^13^C-NMR analyses determined its relevant functional groups, and 2D NMR was used to verify its tetracyclic skeleton and the relative configuration of the substituents. In the assessment of anti-tumor activity, **28** demonstrated significant inhibitory effects across four tumor cell lines. The IC_50_ values of **28** were determined as 3.66 μM for the lung cancer cell line A549, 2.90 μM for the cervical cancer cell line HeLa, 0.03 μM for the prostate cancer cell line DU-145, and 0.10 μM for the liver cancer cell line HepG2. In comparison, the positive control doxorubicin yielded IC_50_ values of 0.01 μM, 0.05 μM, 0.34 μM, and 0.92 μM against the same respective cell lines. Furthermore, **29** and **30** exhibited notably weaker activities, with IC_50_ ranges of 18.70–46.97 μM and 13.20–53.00 μM, respectively, across the tested cell lines. Notably, **28** displayed its strongest inhibitory potency against the prostate cancer cell line DU-145, with an IC_50_ of merely 0.03 μM, which was substantially lower than that of doxorubicin (0.34 μM), highlighting its promising anti-tumor potential. The SAR analysis indicated that the extended tetracyclic skeleton system in **28**′s structure and its terminal aromatic imidazole side chain facilitated its binding to multiple targets, while the introduction of polar functional groups enhanced its affinity and selectivity with the targets.

**Figure 19:**
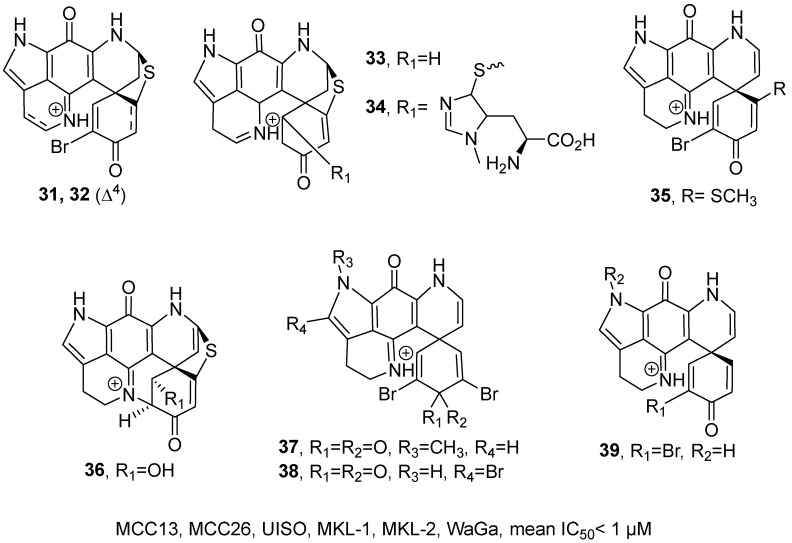
Structures of marine-derived alkaloids discorhabdin derivatives A (**31**), B (**32**), N-13-demethyl U (**35**), P (**37**), 14-Br-discorhabdin C (**38**), L (**36**), E (**39**), G/I (**33**) and **34**.

In 2023, J. Henrich’s research group isolated 18 discorhabdin-type compounds from marine sponges and synthesized six derivatives through semi-synthesis (Figure 19) [60]. All the compounds belong to alkaloids. Taking the structural elucidation of **34** as an example: its molecular formula was established as C_26_H_25_N_6_O_4_S_2_ by HRMS, and the ^1^H NMR spectrum revealed the presence of additional methyl signals, and correlations observed in the ^1^H and ^13^C HMBC spectrum. Ultimately, the absolute configuration of **34** was unequivocally assigned based on ECD analysis. Activity assays revealed that discorhabdin derivatives A (**31**), B (**32**), N-13-demethyl U (**35**), P (**37**), 14-Br-discorhabdin C (**38**), L (**36**), E (**39**), and G/I (**33**) exhibited potent cytotoxicity, with mean IC_50_ values below 1 μM across six tested cell lines, including MCC13, MCC26, UISO, MKL-1, MKL-2, and WaGa. SAR analysis identified the α-bromoalkenone group as the essential pharmacophore. Activity decreased upon modification of lactone ring E or insertion of a C-2-N-14 bridge. Methylation of pyrrole ring A and the C-5-C-8 thioether bridge enhanced potency, while saturation of the C-4-C-5 double bond improved activity but reduced selectivity. Introducing an alkenyl group between C-7 and C-8 or a bulky substituent at C-5 negatively impacted efficacy.

**Figure 20:**
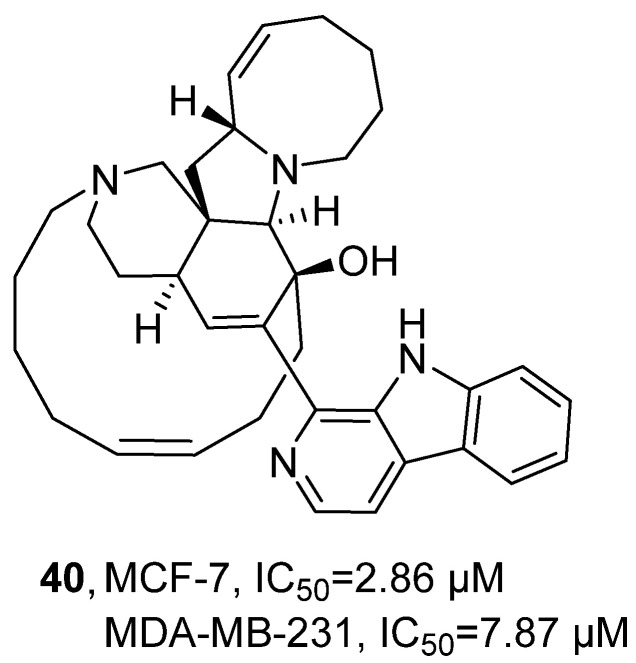
Structure of marine-derived alkaloid Manzamine A (**40**).

In 2023, Han’s research group conducted an activity assessment on the *β*-cycline alkaloid Manzamine A (MA, **40**), which was previously isolated from marine sponges (Figure 20) [61]. Using MTT assay, activity evaluation demonstrated that, compared with the positive control drug paclitaxel, compound **40** exhibited weaker cytotoxic activity against MCF-7 and MDA-MB-231 breast cancer cells, with IC_50_ values of 2.86 μM and 7.87 μM, respectively, while paclitaxel showed IC_50_ values of 0.0157 μM and 0.0017 μM, respectively.

**Figure 21:**
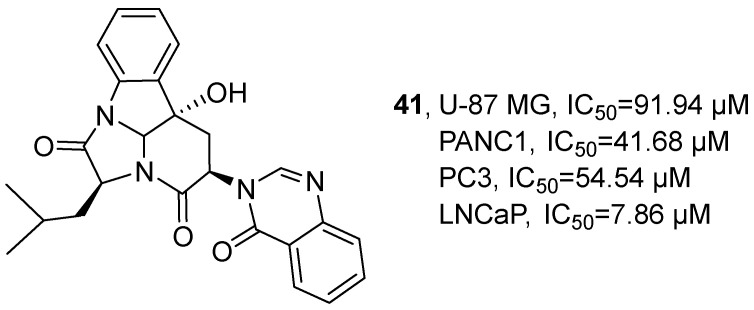
Structure of marine-derived alkaloid isopropylchactominine (**41**).

In 2024, Avci’s research group isolated a pyrimidine alkaloid, isopropylchactominine (**41**), from the ethyl acetate extract of the symbiotic fungus *Aspergillus carneus* (Figure 21) [62]. Its molecular formula was determined to be C_25_H_25_N_4_O_4_ by HRMS, and the specific structure was verified by ^1^H NMR and ^13^C NMR. The activity study showed that the IC_50_ values of **41** in U-87 MG, PANC1, PC3 and LNCaP cells were 91.94 μM, 41.68 μM, 54.54 μM and 7.86 μM, respectively.

In alkaloids, nitrogen-containing heterocycles (pyridine, indole, pyrrolidine, guanidine), amide, and aromatic rings are most frequent. The most potent alkaloids come from sponges (Monanchora, Acanthostrongylophora, Neopetrosia) and marine fungi (Aspergillus, Penicillium). Among them, Meleagrin (**25**) shows an IC_50_ as low as 0.03 μM against DU-145 prostate cancer cells, far better than doxorubicin; Monanchocidin A (**17**) exhibits GI_50_ values of 0.018–0.138 μM against melanoma lines, surpassing paclitaxel. Pentacyclic guanidine core or epidithiodiketopiperazine (ETP)-containing alkaloids display the best activity. Trends from 2020 to 2024 include that structural elucidation by HRMS, 2D-NMR, and X-ray diffraction; identification of pharmacophores (e.g., α-bromoalkenone, disulfide); semi-synthetic optimization; and target prediction revealing multi-target actions on kinases and proteasomes.

### 2.3. Marine-Derived Steroids

Marine-derived sterols, encompassing sulfated sterols, sterol derivatives, and related congeners, are primarily isolated from sponges and other benthic invertebrates. The fundamental sterol scaffold in these compounds frequently undergoes unique structural modifications, including specialized side-chain substitutions, sulfation, activation, or glycosylation, which impart distinct molecular architectures and physicochemical properties relative to terrestrial sterols. This structural diversification, along with their biosynthetic pathways, reflects a pronounced marine chemical specificity, establishing them as a key resource for the discovery of novel anti-tumor lead compounds.

**Figure 22:**
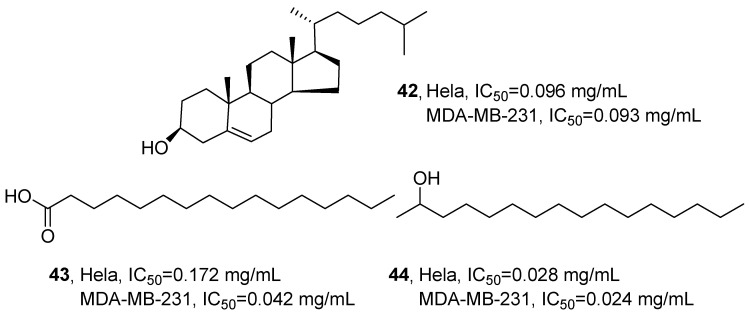
Structures of marine-derived steroids (3*β*)-cholest-5-en-3-ol (**42**), hexadecanoic acid (**43**) and 2-hexadecanol (**44**).

In 2020, Niknejad’s research group isolated three known compounds from the ethyl acetate extract of *Virgularia gustaviana*: a steroidal constituent, (3*β*)-cholest-5-en-3-ol (**42**); a fatty acid, hexadecanoic acid (**43**); and a fatty alcohol, 2-hexadecanol (**44**) (Figure 22) [63]. Taking the structural identification of **42** as an example, by comprehensively applying ^1^H NMR, ^13^C NMR, 2D NMR (including ^1^H−^1^H COSY, HMQC, HMBC), MS and UV analysis techniques, and analyzing the absorption at 270 nm, the data were compared with the former reports to confirm that this compound was **42**. Bioactivity evaluation revealed that **42** displayed significant cytotoxicity against HeLa and MDA-MB-231 cells, with IC_50_ values of 0.096 mg/mL and 0.093 mg/mL, respectively. **43** showed weaker activity against HeLa cells (IC_50_ = 0.172 mg/mL) but enhanced potency against MDA-MB-231 cells (IC_50_ = 0.042 mg/mL). Notably, **44** exhibited the strongest inhibitory effect on both cell lines, demonstrating IC_50_ values of 0.028 mg/mL (HeLa) and 0.024 mg/mL (MDA-MB-231).

**Figure 23:**
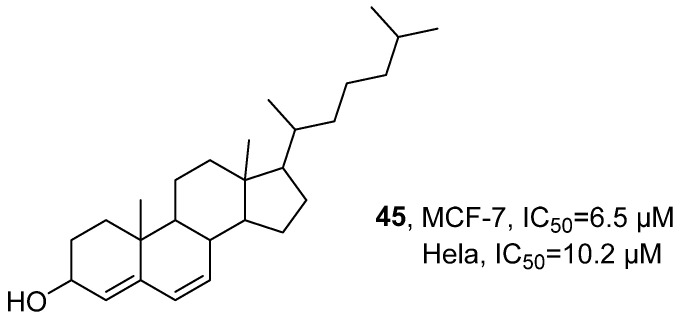
Structure of marine-derived steroid cholesta-4,6-dien-3-ol (**45**).

In 2021, Zaki’s research group isolated multiple components from a dichloromethane extract of the sea squirt *Eudistoma kaverium*, which is from the waters of India (Figure 23) [64]. These isolates included steroidal constituents such as cholesta-4,6-dien-3-ol (EK-7, **45**). Taking **45** as an example, a comprehensive structural analysis was conducted using techniques such as HPLC, GC-MS, and HRMS to infer its molecular structure and determine the molecular formula as C_27_H_44_O. Cytotoxicity assessment revealed that **45** demonstrated potent activity against both the breast cancer cell line MCF-7 and the cervical cancer cell line HeLa, with respective IC_50_ values of 6.5 μM and 10.2 μM.

**Figure 24:**
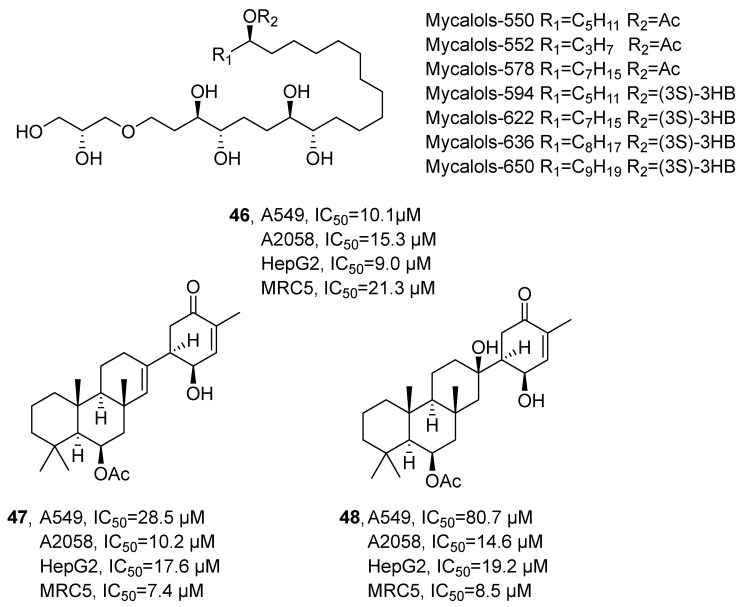
Structures of marine-derived steroids mycalols (**46**), suberitenones A (**47**) and B (**48**).

In 2021, Lauritano’s research group isolated mycalols (**46**), suberitenones A (**47**) and B (**48**) from the methanol extracts of the sponges *Hemimycale topsenti* and *Haliclona (Rhizoniera) dancoi* (Figure 24) [65]. The compounds were structurally characterized as an alkyl ether and a terpenoid, respectively, based on NMR and HRMS analyses. Cytotoxicity evaluation revealed that **46** exhibited potent anti-tumor activity against A549, A2058, HepG2, and MRC5 cell lines, with IC_50_ values of 10.1, 15.3, 9.0, and 21.3 μM, respectively. In comparison, **47** showed IC_50_ values of 28.5, 10.2, 17.6, and 7.4 μM, while **48** displayed IC_50_ values of 80.7, 14.6, 19.2, and 8.5 μM against the same panel of cell lines. SAR analysis indicated that cytotoxic potency correlates with alkyl chain length and the nature of substituents.

### 2.4. Marine-Derived Polyketides

Marine-derived polyketides are precisely synthesized by the modular mechanism of polyketide synthases, feature diverse polycyclic, aromatic, fatty chain, and heterocyclic frameworks with high structural complexity in oxidation states, substitution patterns, and chiral centers. Leveraging the biosynthetic capacity of marine microorganisms and their symbionts, these metabolites greatly expand the accessible natural product chemical space. Their structural complexity and scaffold diversity make them a key class in natural product-based lead discovery for developing novel anticancer therapeutics.

**Figure 25:**
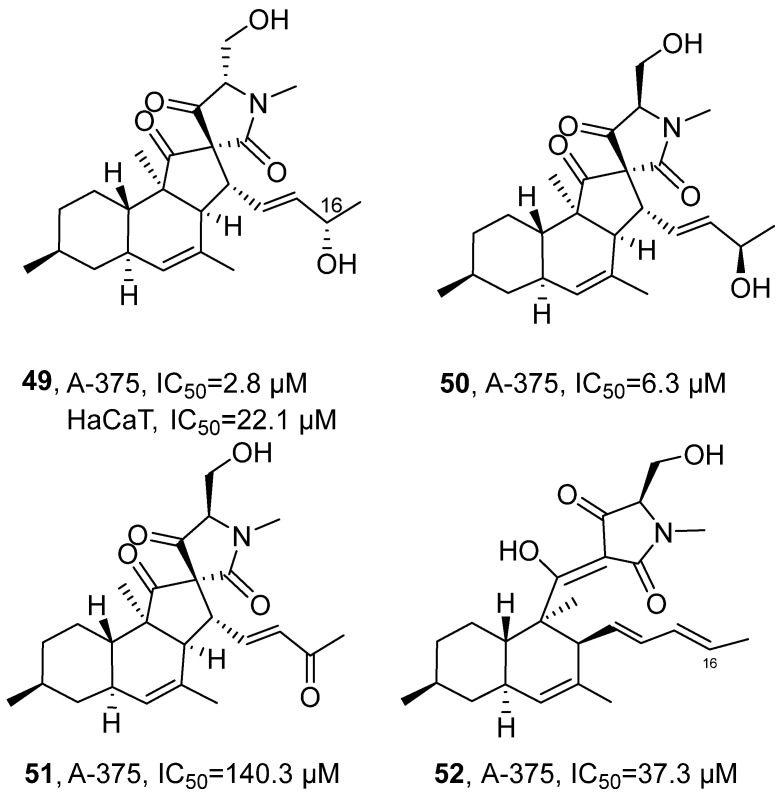
Structures of marine-derived polyketides pyrenosetins A-C (**49–52**).

In 2020, Tasdemir’s research group isolated three new decalinoylspirotetramic acid derivatives, pyrenosetins A-C (**49–51**), from the ethyl acetate extract of the endophytic fungus *Pyrenochaetopsis* sp. FVE-001 of the brown alga *Fucus vesiculosus* (Figure 25) [66], along with the known compound phomasetin (**52**), all belonging to the polyketide class. Taking the structural analysis of **49** as an example, its molecular formula was determined by HRMS as C_25_H_35_NO_5_, corresponding to nine degrees of unsaturation. FT-IR spectrum indicated the presence of hydroxyl and carbonyl groups in the structure. The ^1^H NMR and DEPT-HSQC spectra displayed five methyl signals including N-methyl. The ^13^C NMR spectrum further revealed three carbonyl carbon signals. Through COSY correlation signals, three distinct spin systems were identified, while HMBC correlation substantiated the connectivity between the decalin and spirocyclic ring systems. Finally, application of the Mosher ester method established the absolute configuration at C-16 as *S*. Compared with the positive control drug doxorubicin (IC_50_ = 0.6 μM against A-375 and 22.1 μM against HaCaT), compound 49 exhibited weaker activity against A-375 cells (IC_50_ = 2.8 μM) but stronger activity against HaCaT cells (IC_50_ = 4.2 μM); compound 50 showed weaker activity against A-375 cells (IC_50_ = 6.3 μM); and compounds 51 and 52 exhibited considerably weaker activity against A-375 cells (IC_50_ = 140.3 μM and 37.3 μM), respectively. SAR analysis indicated that both the stereochemical configuration and the oxidation state at C-16 critically influence biological activity. The presence of a hydroxyl group at C-16 was associated with enhanced potency; however, variations in stereoconfiguration at this position markedly affected compound selectivity and toxicity, compounds **49** with 16*S* configuration and compound **50** with 16*R* configuration exhibit higher anti-tumor activity, while compounds **51** with a planar carbonyl structure at C-16 and compounds **52** with a planar carbon–carbon double bond have lower anti-tumor activity; the overall toxicity of the isolated compounds was evaluated using the human keratinocyte cell line HaCaT, and 16*R* configuration can effectively reduce the toxicity of the compounds. Conversely, oxidation of the C-16 hydroxyl to a ketone group resulted in a significant reduction in anti-proliferative activity.

**Figure 26:**
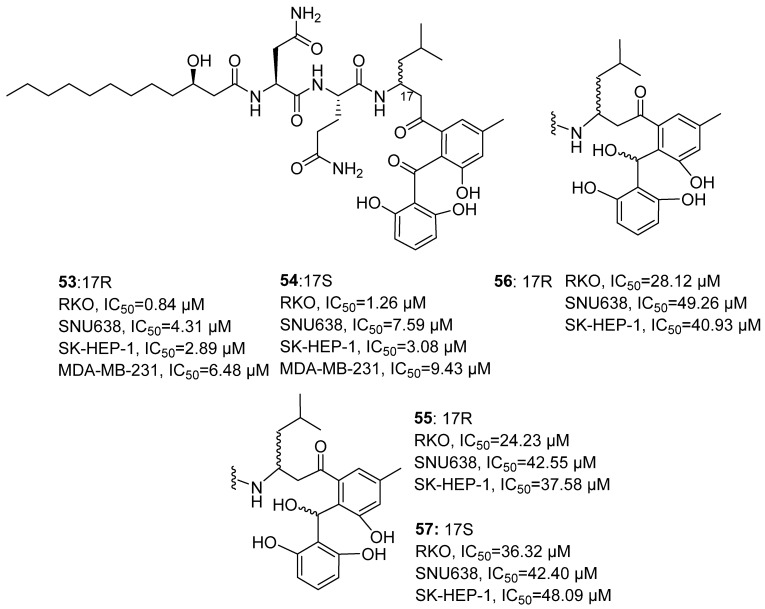
Structures of marine-derived polyketides Asperphenin A (**53**), Asperphenin B (**54**) and hydroxylated derivatives (**55–57**).

In 2020, Lee’s research group isolated Asperphenin A (**53**) from the dimethylformamide extract of marine-derived *Aspergillus* sp. (Figure 26) [67]. **53** is a structurally novel lipopeptidyl phenol ketone-type natural product. The research group had previously synthesized 10 derivatives of **53**. Taking **55** as an example, its structure was confirmed through nuclear magnetic resonance ^1^H NMR and ^13^C NMR. Combined with the MTPA esterification method, coupling constants (J) analysis, and ECD calculation, the absolute configuration of the trichothecene and its synthetic derivatives at all chiral centers was characterized. Evaluation of in vitro anti-tumor activity showed that **53** exhibited the most significant activity, with IC_50_ values of 0.84, 4.31, 2.89, and 6.48 μM against four human tumor cell lines (RKO, SNU638, SK-HEP-1, MDA-MB-231). Asperphenin B (**54**) showed slightly lower activity than **53**, with corresponding IC_50_ values of 1.26, 7.59, 3.08, and 9.43 μM. The positive control drug Etoposide, with IC_50_ values of 3.86, 0.30, 0.49, and 10.72 μM, showing pronounced activity in SNU638 cells. The hydroxylated derivatives (**55–57**) generally exhibited diminished inhibitory activity, with IC_50_ values ranging from 24.23 to 49.26 μM. SAR analysis revealed that the aryl ketone group at the C-7 position is a crucial pharmacophore for maintaining activity. Reduction of this group to a hydroxyl or alkene led to loss of activity, while other structural modifications also resulted in a significant decrease in potency.

**Figure 27:**
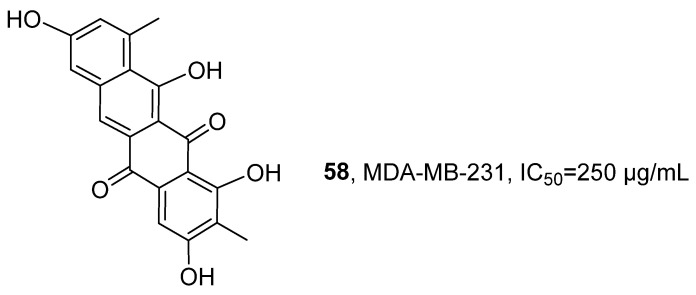
Structure of marine-derived polyketide Persiamycin A (**58**).

In 2020, Metsä-Ketelä’s research group isolated a new compound, Persiamycin A (**58**), from the ethyl acetate and methanol extracts of the halophilic actinobacterium *Streptomonospora* sp. PA3 (Figure 27) [68]. This compound belongs to the aromatic polyketide class. Its structure was confirmed through the following methods: HRMS determined its molecular formula to be C_20_H_13_O_6_; the ^1^H NMR spectrum revealed four aromatic proton signals, two methyl signals, and three partially interchangeable hydroxyl signals; HMBC analysis further confirmed the correlation between the methyl group and the phenolic carbon, indicating the presence of methyl and hydroxyl substitution on the aromatic ring. The activity evaluation results showed that Persiamycin A exhibited relatively weak anti-proliferative activity against the human breast cancer cell line MDA-MB-231 in vitro, with an IC_50_ value of 250 µg/mL.

**Figure 28:**
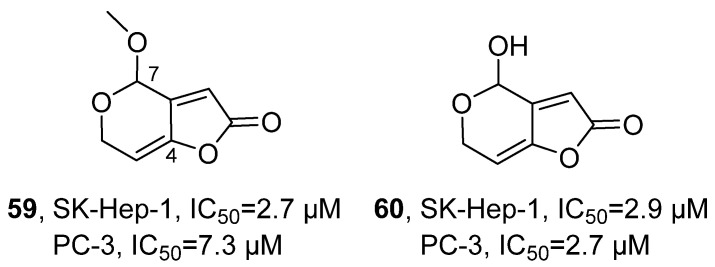
Structures of marine-derived polyketides aspergilsmin C (**59**) and patulin (**60**).

In 2020, Lee’s research group isolated aspergilsmins A-G from the ethyl acetate extract of the *Aspergillus giganteus* NTU967 fungus, which were all identified as polyketide compounds (Figure 28) [69]. Among them, aspergilsmin C (**59**) was confirmed to be a methyl derivative of patulin (**60**). Taking aspergilsmin C as an example for structure elucidation, its molecular formula was determined to be C_8_H_8_O_4_, with five unsaturations. NMR- and HMBC-related signals further confirmed that the methyl and ethyl groups were connected at the C-7 position. The activity evaluation results showed that compound 3 had an IC_50_ value of 2.7 μM in SK-Hep-1 liver cancer cells and 7.3 μM in PC-3 prostate cancer cells. And compared with the positive control drug paclitaxel, patulin exhibited weaker cytotoxic activity against SK-Hep-1 and PC-3 cells, with IC_50_ values of 2.9 μM and 2.7 μM, respectively, while paclitaxel showed IC_50_ values of 0.011 μM and 0.013 μM, respectively. The SAR analysis revealed that the size of the substituent at the C-7 position and the olefin structure at the C-4 position are crucial for maintaining the anti-tumor activity of this class of compounds.

**Figure 29:**
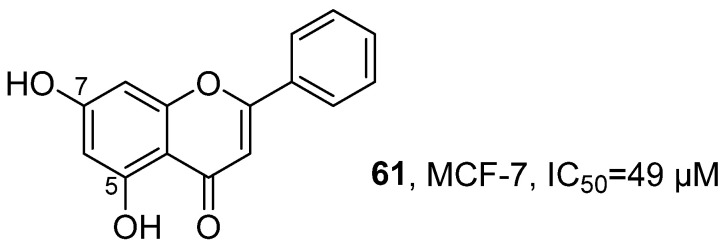
Structure of marine-derived polyketide Chrysin (5,7-dihydroxyflavone) (**61**).

In 2020, Jayabaskaran’s research team isolated Chrysin (5,7-dihydroxyflavone) (**61**) from the ethyl acetate extract of the marine endophytic fungus *Chaetomiumglobosum* (Figure 29) [70]. This compound belongs to the polyketide class. Its structure was confirmed through various spectroscopic techniques: The IR spectrum revealed the presence of O-H, C = O, C-CO-C and C-O-C functional groups; LC-MS analysis established its molecular formula to be C_15_H_10_O_4_; and the NMR spectrum further clarified that the hydroxyl groups were located at C-5 and C-7 positions. Using MTT and resazurin reduction assay, the activity evaluation results showed that Chrysin (5,7-dihydroxyflavone) exhibits significant anti-tumor activity, with an IC_50_ value of approximately 49 µM against human breast cancer cells MCF-7.

**Figure 30:**
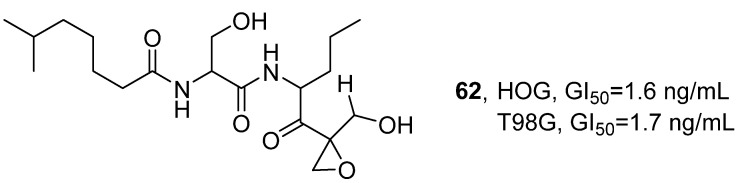
Structure of marine-derived polyketide dihydroeponemycin (DHE, **62**).

In 2021, V. Costa-Lotufo’s research group isolated a variety of polyketide cyclic compounds, including dihydroeponemycin (DHE, **62**), from the marine actinomycete BRA-346 that coexists with the Brazilian endemic ascidian *Euherdmania* sp. Through extraction and separation with ethyl acetate, a total of 13 eponemycin analogs were obtained (Figure 30) [71]. Taking DHE as an example for structure confirmation, the ion peak *m*/*z* 401.26 was detected through HPLC-MS/MS analysis, which was consistent with the known structure. The activity evaluation results showed that when the incubation time of DHE and the BRA-346 containing epoxy ketone components was extended from 24 h to 48 h, the growth inhibitory activity increased 10-fold; however, further extension to 72 h did not lead to additional enhancement. Both cell lines exhibited similar sensitivity to DHE, with the GI_50_ values at 48 h being 1.6 ng/mL and 1.7 ng/mL in HOG and T98G cells, respectively. In contrast, T98G cells were slightly less sensitive to the epoxyketone-containing fraction of BRA-346, with a GI_50_ of 28.2 ng/mL, while that of HOG cells was 17.6 ng/mL. The SAR analysis indicated that the epoxy ketone structure was the key pharmacophore for inhibiting proteasome activity; moreover, synergistic effects among the various structural analogs present in the BRA-346 fraction may further enhance its biological activity.

**Figure 31:**
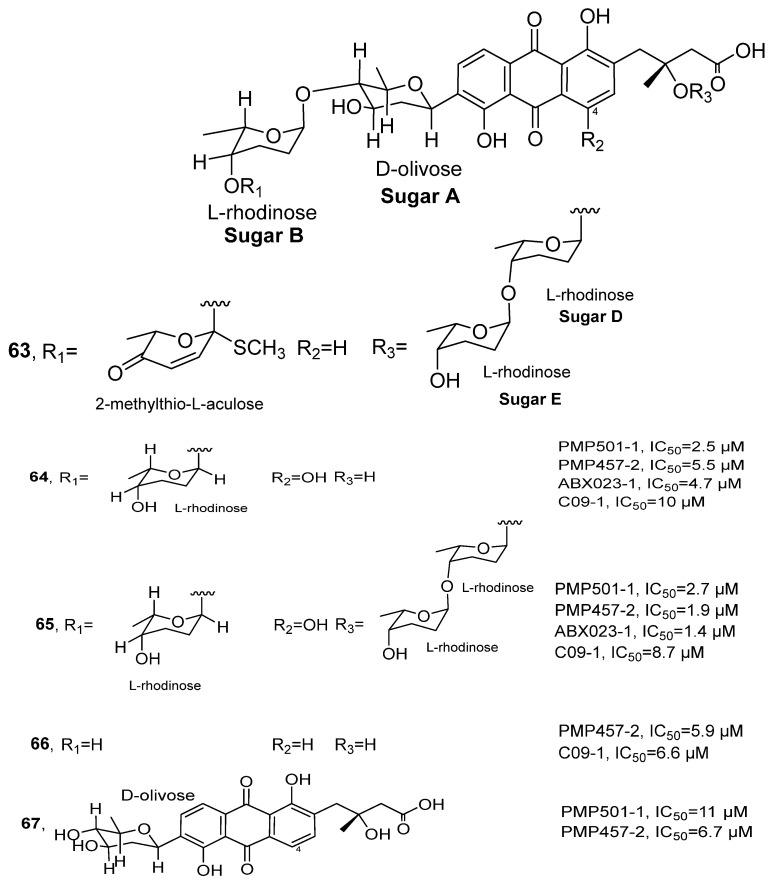
Structures of marine-derived polyketides Grincamycin R (**63**) and **64–67**.

In 2021, Li’s research group isolated a series of rearranged angucycline compounds Grincamycins P-T from the ethyl acetate extract of *Streptomyces* sp. CNZ-748 (Figure 31) [72], which originated from marine sediments. These compounds belong to the polyketide family. Taking Grincamycin R (**63**) as an example for structure identification: HRMS showed its molecular formula as C_50_H_64_O_18_S and NMR data further confirmed its planar structure. Combined with HMBC and COSY correlation signals, a novel *α*-L-methylthio-akulose residue was inferred in its structure. The stereochemistry was ultimately determined by integrating NOE correlation data and biosynthetic pathway analysis. The activity evaluation results showed that compound **65** exhibited the optimal inhibitory activity in four tested cell lines, with IC_50_ values of 2.7, 1.9, 1.4 and 8.7 μM for PMP501-1, PMP457-2, ABX023-1 and C09-1, respectively. Its activity was superior to the positive control drug 5-fluorouracil in three cell lines. Compound **64** also displayed broad-spectrum inhibitory activity, with IC_50_ values ranging from 2.5 to 10 μM. Additionally, compounds **66** and **67** showed moderate activity for some cell lines, with IC_50_ values ranging from 5.9 to 11 μM. The SAR analysis suggested that the hydroxylation at the C-4 position and the introduction of a methylthio group in the terminal trisaccharide chain might significantly enhance cytotoxicity, potentially by improving solubility or strengthening interactions with target sites.

**Figure 32:**
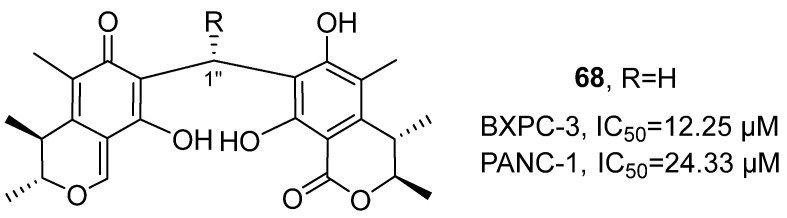
Structures of a marine-derived polyketide **68**.

In 2022, Zhang’s research group isolated four novel carbon-linked citrinin dimeric compounds from the ethyl acetate extract of the symbiotic fungus *Penicillium* sp. GGF16-1-2 of sea stars (Figure 32) [73], all of which belong to the polyketide structural class. Taking the structure of compound **68** as an example: Through HRMS, its molecular formula was determined as C_25_H_28_O_7_. HMBC and COSY correlation signals confirmed that the two structural fragments were connected by a carbon bridge (C-1″). The relative configuration was deduced by NOESY spectroscopy, and the absolute configuration (3*R*, 4*S*, 3′*R*, 4′*S*) was confirmed by comparison of experimental and calculated ECD spectra. Based on ^1^H NMR and ^13^C NMR data, it can be inferred that its structure contains a ketone carbonyl and an ester carbonyl. The in vitro cell activity evaluation results showed that, compared with the positive control drug doxorubicin (IC_50_ = 18.24 µM for BXPC-3 and 24.00 µM for PANC-1), compound 66 exhibited stronger cytotoxic activity against BXPC-3 and PANC-1 cells, with IC_50_ values of 12.25 µM and 24.33 µM, respectively.

**Figure 33:**
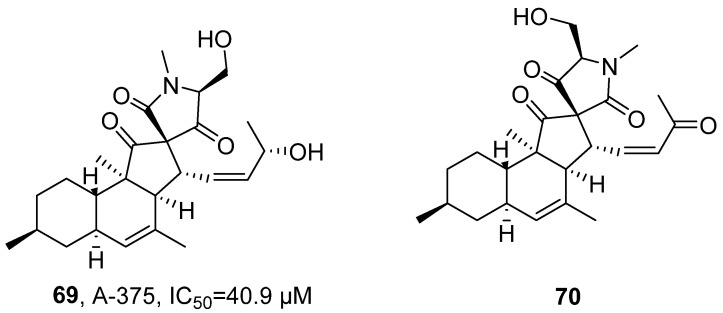
Structures of marine-derived polyketides Pyrenosetin E (**69**) and Pyrenosetin F (**70**).

In 2022, Tasdemir’s research team isolated two endophytic fungi, *Pyrenochaetopsis* sp. FVE-001 and FVE-087, from the brown alga Fucus vesiculosus (Figure 33) [74]. After extraction with ethyl acetate, tenhydronaphthalenyl lactone tetracyclic acid derivatives Pyrenosetin E (**69**) and Pyrenosetin F (**70**) belonging to polyketide compounds were obtained. Taking Pyrenosetin E as an example, its molecular formula was determined as C_25_H_35_NO_5_ by HRMS. The structure was confirmed by FT-IR to contain carbonyl and hydroxyl groups. Further analysis using NMR (^1^H-NMR, ^13^C-NMR) revealed that the compound has four alkenes, three carbonyls, and five unsaturation degrees, indicating a tetracyclic structure. Finally, combined with 2D NMR data such as COSY, DEPT-HSQC, and HMBC, the chemical structure was further verified and determined. The activity evaluation results showed that Pyrenosetin E has inhibitory activity against human malignant melanoma cell line A-375, with an IC_50_ value of 40.9 μM.

**Figure 34:**
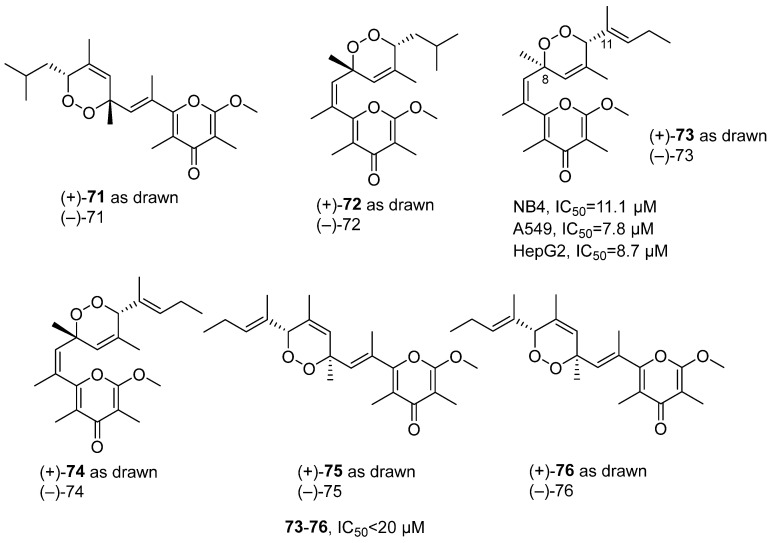
Structures of marine-derived polyketides (±)-Ocellatuperoxides A-F (**71–76**).

In 2022, Guo’s research group isolated six new pairs of γ-pyrone polypropionate enantiomers with an unusual peroxyl bridge at the side chain, from the MeOH-CH_2_Cl_2_ extract of the *Placobranchus ocellatus*, namely (±)-Ocellatuperoxides A-F (**71–76**) (Figure 34) [75]. Structural elucidation of (±)-Ocellatuperoxide C was determined by HRMS, with molecular formula C_22_H_30_O_5_. The unsaturated carbonyl group was determined by infrared spectroscopy, and the existence of an endoperoxide bridge between C-8 and C-11 was confirmed by NMR, COSY, and HMBC, forming a 1,2-dioxane ring connection between the fragments. In vitro activity tests showed that the compound (±)-Ocellatuperoxides C-F had inhibitory activity against cancer cells, with IC_50_ values all within the range of 20 μM. Among them, (±)-Ocellatuperoxides C had the best activity, with IC_50_ values of 11.1, 7.8, and 8.7 μM for NB4, A549, and HepG2 cells respectively. The preliminary SAR indicated that the activity was weaker when the side-chain terminal was isobutyl, while compounds with a 1-methyl-1-butenyl side chain had stronger activity, and the stereoconfiguration had a significant impact on activity; (±)-compound **73** was evaluated on the A549 cell line. Interestingly, the results indicated that only (−)-compound **73** (IC_50_ = 8.7 μM) exhibited activity, while (+)-compound **73** (IC_50_ > 100 μM) was inactive.

**Figure 35:**
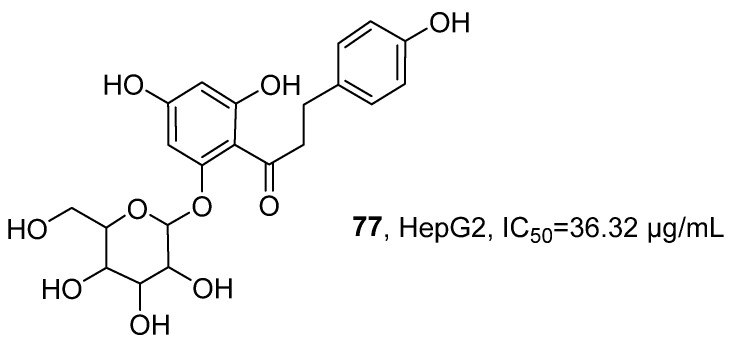
Structure of marine-derived polyketide phloridzin (**77**).

In 2023, Sanniyasi’s research group successfully isolated the known dihydrochalcone glycoside compound phloridzin (**77**) from the ethanol–water extract of the sea grass *Syringodium isoetifolium* (Figure 35) [76]. The molecular formula was determined to be C_21_H_24_O_10_ by MS. Further analysis using various spectroscopic techniques such as FT-IR, ^1^H NMR, and ^13^C NMR confirmed its chemical structure. The activity evaluation results showed that, compared with the positive control drug doxorubicin, compound **77** exhibited weaker cytotoxic activity against HepG2 hepatocellular carcinoma cells, with an IC_50_ value of 36.32 μg/mL, while doxorubicin showed an IC_50_ value of 10.19 μg/mL.

In polyketides, aromatic rings, lactone rings, epoxyketone, methoxy, and glycosyl groups are most frequent. The most potent polyketides derive from marine fungi (Pyrenochaetopsis, Aspergillus, Penicillium) and actinomycetes (Streptomyces). Asperphenin A (**53**) shows an IC_50_ of 0.84 μM against RKO colon cancer cells, comparable to etoposide; grincamycin derivative (**65**) exhibits IC_50_ as low as 1.4–2.7 μM against rare cancer lines. Spirotetramic acid-, epoxide-, or carbon-bridged dimer-containing polyketides display the best activity. Trends from 2020 to 2024 include that targeted isolation via feature-based molecular networking; decisive role of absolute configuration (e.g., only (−)-ocellataperoxide C is active); identification of key pharmacophores (C-7 aryl ketone, epoxyketone); and development of polyketides as payloads for antibody-drug conjugates (ADCs).

### 2.5. Marine-Derived Peptides and Proteins

Marine-derived peptides and proteins constitute a highly diverse family in terms of sequence, spatial conformation, and modification patterns. These molecules are often rich in disulfide bonds, undergo glycosylation, or carry other complex post-translational modifications. These molecules are widely distributed in sponges, marine microorganisms, and their symbiotic systems, and frequently contain characteristic motifs such as non-canonical amino acids and unique cyclic backbones. This unique structure endows them with outstanding advantages in molecular recognition ability, conformational stability, and plasticity, making marine peptides a highly promising source of lead compounds in medicinal chemistry and providing a rich array of molecular scaffolds for the development of novel anticancer drugs.

In 2020, R. O’Keefe’s research group conducted an activity substance screening on the marine sponge *Axinella* sp. and discovered a new type of cyclic peptide named Recifin A, which is rich in cysteine [77]. This peptide consists of 42 amino acid residues and belongs to the structurally novel Tyr-lock family. The research team determined its primary sequence through MS/MS and automatic Edman degradation, and elucidated its three-dimensional structure using NMR technology. Based on 425 distance constraints and 75 dihedral angle constraints, the core structure was defined as a four-chain *β*-sheet flanked by two helical turns; the disulfide bonds formed a typical cysteine knot, locking Tyr6 into a specific spatial conformation. Further partial reduction and alkylation experiments clarified that the disulfide connectivity was Cys I–III, II–V, and IV–VI. The activity study showed that Recifin A could effectively inhibit tyrosine-DNA phosphodiesterase 1 (TDP1), with an IC_50_ value of 190 nM.

**Figure 36:**
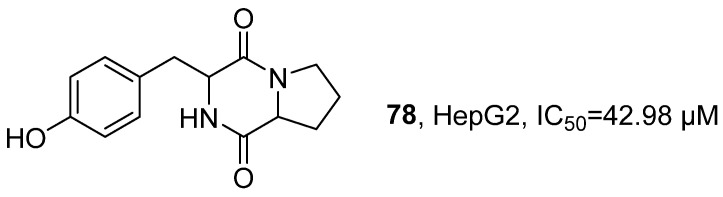
Structure of marine-derived peptide component F2 (**78**).

In 2020, Karanam’s research group isolated 11 components from the ethyl acetate extract of *Bacillus pumilus* co-associated with marine sponges (Figure 36) [78]. Through preliminary TLC screening, component F2 (Cyclo(-Pro-Tyr), **78**) showed high biological activity and belonged to a polypeptide compound. Using techniques such as LC-MS/MS, FT-IR, and NMR, the structure was systematically analyzed: HRMS showed its parent ion peak at *m*/*z* 261.07 [M + H]^+^, suggesting its molecular formula as C_14_H_16_N_2_O_3_; IR confirmed the presence of amide carbonyl (C = O), N-H, and O-H stretching vibrations in the structure; NMR spectrum further revealed signals corresponding to a benzene ring and two carbonyl groups. Using MTT assay, the activity evaluation results indicated that **78** had significant cytotoxicity against HepG2 human hepatocellular carcinoma cells, with an IC_50_ value of 42.98 μM.

**Figure 37:**
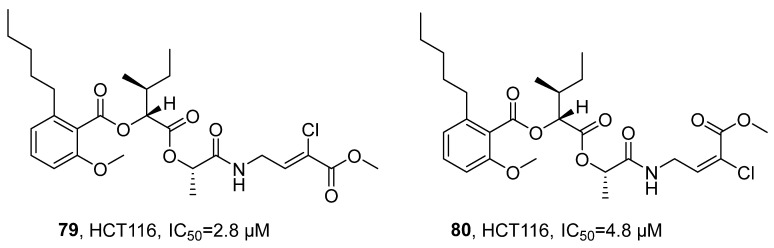
Structures of marine-derived peptides anaenamides A (**79**) and B (**80**).

In 2020, Luesch’s research group isolated a new class of peptide natural products anaenamides A (**79**) and B (**80**) from the marine cyanobacterium *Hormoscilla* sp. (Figure 37) [79]. Taking anaenamide A as an example, its molecular formula was determined to be C_27_H_39_NO_8_Cl by HRMS. The structure was analyzed using multi-dimensional NMR techniques: the HSQC spectrum indicated the presence of six methines, three methylenes, four methyl groups, three consecutive aromatic proton signals, one isolated olefin proton, and two methoxy signals; the ^13^C NMR spectrum displayed the presence of four unprotonated sp^2^ hybrid carbons and four carbonyl carbon signals. By further combining COSY and HMBC correlation spectra, three structural fragments, alkyl salicylate, 2-hydroxy-3-methylpentanoic acid, and lactate, were identified. Their planar structures and relative configurations were ultimately confirmed, with the assistance of HMBC and NOESY spectra. The activity study showed that anaenamides A and B exhibit significant inhibitory activity against human colon cancer cells HCT116, with IC_50_ values of 2.8 μM and 4.8 μM, respectively. Preliminary SAR analysis suggested that the halogenated *α*,*β*-unsaturated ester unit acts as a Michael acceptor, as its removal or saturation led to a significant reduction in cytotoxicity, indicating that this moiety is the key pharmacophore responsible for the cytotoxic activity.

In 2020, Giovine’s research group isolated and purified a new protein from the extruded crude extract of the sponge *Chondrosia reniformis* through a series of steps including 10 kDa filtration dialysis, ammonium sulfate precipitation, and high-performance liquid chromatography size exclusion chromatography [80]. MS analysis revealed that the protein precursor consisted of 199 amino acids, including a 21-amino acid signal peptide and a 178-amino acid mature peptide, with a theoretical molecular weight of 19,611.12 Da and a theoretical isoelectric point was 5.11. Sequence alignment results indicated that this protein exhibits structural homology with the N-terminal region of the ryanodine receptor and defensin-like proteins. The research further completed the three-dimensional structural modeling of the protein using Phyre2. The activity evaluation results showed that the purified components Chondrosin P4 and P5 exhibited differential activities on various cell lines: the EC_50_ for RAW264.7 cells was 1.99 μg/mL and 1.12 μg/mL, respectively; for L929 cells, the EC_50_ was 3.56 μg/mL and 4.90 μg/mL, respectively; for MDA-MB-468 cells, the EC_50_ of P4 was 59.88 μg/mL; for HeLa cells, the EC_50_ of P4 was 17.7 μg/mL; and for normal human dermal fibroblasts, the EC_50_ of P5 and P4 were 14.6 μg/mL and 17.8 μg/mL, respectively.

**Figure 38:**
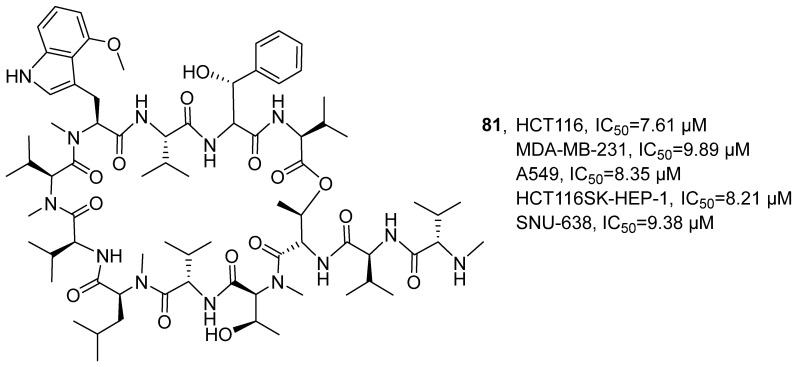
Structure of marine-derived peptide Ohmyungsamycin A (**81**).

In 2020, building upon previous studies, Lee’s research group conducted further research on a novel cyclic peptide named Ohmyungsamycin A (**81**), which was isolated from the marine bacterium SNJ042 of *Streptomyces* genus (Figure 38) [81]. The activity evaluation results showed that, compared with the positive control drug etoposide, compound **81** exhibited weaker cytotoxic activity against MDA-MB-231, HCT116, A549, SK-HEP-1, and SNU-638 cells, with IC_50_ values of 9.89, 7.61, 8.35, 8.21, and 9.38 μM, respectively, while etoposide showed IC_50_ values of 3.85, 0.52, 0.48, 0.78, and 0.82 μM, respectively.

**Figure 39:**
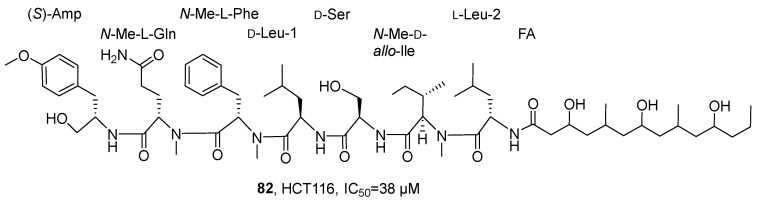
Structure of marine-derived protein wenchangamide A (**82**).

In 2021, Luzzatto-Knaan’s research group isolated and identified a lipopeptide compound, wenchangamide A (**82**), from the dichloromethane and methanol extracts of cf. *Neolyngbya* cyanobacteria from the South China Sea (Figure 39) [82]. For the structural analysis of wenchangamide A, the study initially characterized it using LC-MS/MS technology and further determined the absolute configuration of its amino acid residues by the Marfey method. In vitro activity evaluation showed that this compound could concentration-dependently induce apoptosis in HCT116 human colon cancer cells, with a 24 h IC_50_ value of 38 μM. The SAR analysis revealed that the core peptide backbone, such as the N-methylphenylalanine residue, might constitute the key pharmacophore, while fatty acid chain length, such as the extended segment in wenchangamide B, might have a regulatory effect on its activity intensity and solubility; the structural differences from mimamide A further indicated that specific domains might have a significant impact on the specificity of the compound’s activity.

In 2021, Zhang’s research group extracted sea cucumber intestinal peptides (SCIP) from the intestinal tissues of sea cucumbers through alkaline protease hydrolysis [83]. HPLC analysis showed that SCIP consisted of a series of oligopeptides. Further analysis revealed that 98.41% of SCIP has a relative molecular mass of less than 2000 Da, and approximately 95.36% of SCIP is distributed within a molecular weight range of less than 1041 Da. The amino acid composition analysis results indicated that SCIP is rich in hydrophobic amino acids and branched amino acids. In vivo zebrafish experiments demonstrated that SCIP reduced MCF-7 fluorescence intensity in a dose-dependent manner when administered via a water-soluble route. The results of in vitro experiments showed that SCIP could dose-dependently inhibit the proliferation of MCF-7 cells and promote their apoptosis at concentrations of 27.8, 83.3, and 250 μg/mL.

**Figure 40:**
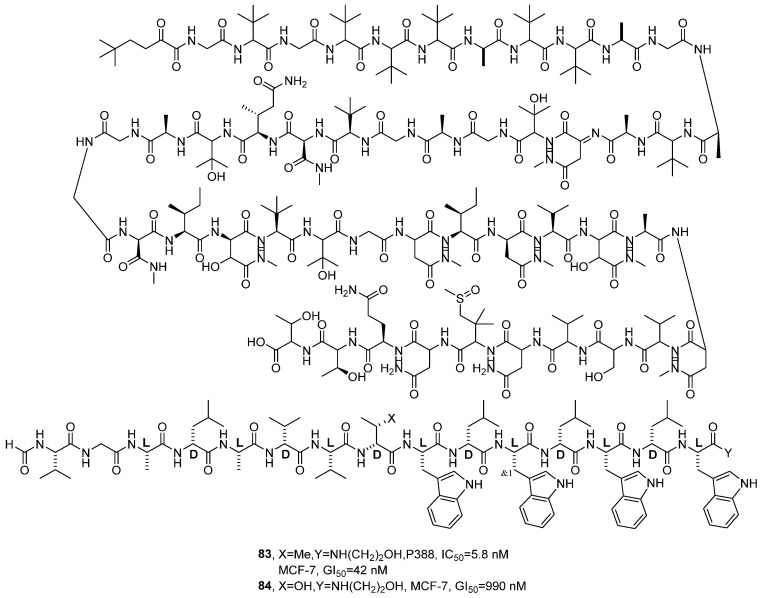
Structure of marine-derived proteins Gramicidin A (**83**) and **84**.

In 2022, Inoue’s research group further explored the mechanism of action of Gramicidin A (**83**), a natural ion channel-forming peptide, based on previous studies by others (Figure 40) [84]. The research team synthesized derivatives 2–4, which are all polypeptides, and their structures were confirmed through key steps in the synthesis route and standard purification methods. The activity detection results showed that, compared with the inactive control drug (**84**), compound **83** exhibited stronger cytotoxic activity against P388 murine leukemia cells, with a GI_50_ value of 42 nM, while the inactive control compound showed a GI_50_ value of 990 nM. The SAR analysis revealed that analog 2, due to a minor structural modification at residue 8 (Me→OH), led to a significant reduction in biological activity (GI_50_ = 990 nM), thereby affecting its ion channel depolarization capacity.

In 2022, Yu’s research group used the hemichordate *Arca inflata Reeve*’s hemolymph as the research subject [85]. Through multi-step of separation and purification combined with an activity-oriented screening strategy, a novel lysine-rich polypeptide P6 was obtained, with a molecular weight of 2794.8 Da. The complete amino acid sequence of this peptide was analyzed using tandem mass spectrometry as WYIRKIRRFFKWLKKKLKK. Circular dichroism spectroscopy analysis revealed that the secondary structure of this peptide segment was mainly α-helix. Three-dimensional structure simulation further indicated that P6 has typical amphiphilic characteristics, forming a positively charged hydrophilic surface and a hydrophobic region. In vitro anti-tumor activity evaluation results showed that the IC_50_ values of P6 against colorectal cancer cells DLD-1, HT-29, and HCT116 were 2.14, 4.43, and 10.88 μg/mL, respectively; although its activity was lower than the positive control cisplatin (with corresponding IC_50_ values were 1.06, 1.90, and 1.38 μg/mL), it still exhibited significant inhibitory effects. In the HT-29 xenograft model, compound P6 demonstrated tumor inhibition rates of 72.66% at a dosage of 30 mg/kg and 49.46% at 15 mg/kg, both superior to the positive control 5-fluorouracil (25 mg/kg, with an inhibition rate of 67.28%), indicating its promising in vivo anti-tumor potential.

**Figure 41:**
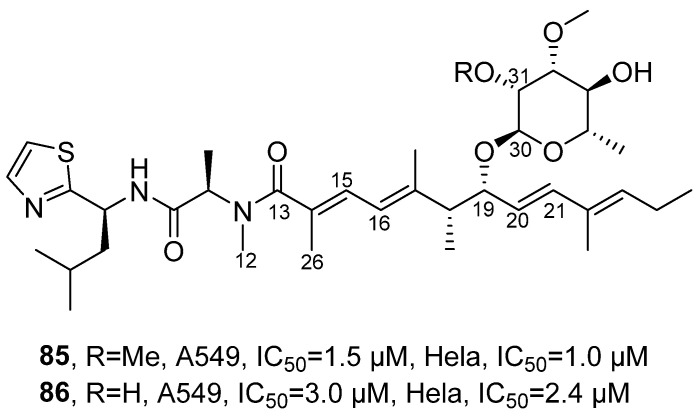
Structure of marine-derived peptides jezoside (**85**) and jezoside B (**86**).

In 2023, Luesch’s research group isolated jezoside (**85**) and its novel derivative jezoside B (**86**) from the cyanobacterial *assemblage* collected in Florida (Figure 41) [86]. Both are polypeptide compounds. For the structural analysis of jezoside B, HRMS determined its molecular formula as C_36_H_57_N_3_O_7_S, identifying it as a similar compound to jezoside with demethylation at C-31. Through COSY and HMBC related signals, its tripeptide unit was determined to be Thz-Leu-N-Me-Ala, where the NMR signals of the N-Me-Ala residue were broadened due to slow conformational exchange. The polyketide unit contained a trans double bond (H-20/H-21) and a conjugated diene (H-15/H-16). With the aid of HMBC related signals, it was further revealed that the tripeptide unit and the polyketide unit were connected through H-26 and H-12 with C-13; the sugar unit was 2,3-O-dimethyl-methylglucoside, and it was connected to the polyketide unit through H-30. Based on J coupling constants, its configuration was determined as s-trans. Activity detection results showed that jezoside and its novel derivative jezoside B had IC_50_ values of 1.5/3.0 µM and 1.0/2.4 µM against A549 and HeLa cells. SAR analysis indicated that the methoxy group at the C-31 position of the sugar moiety enhanced the activity but was not an essential structural unit. Removal of this group only caused a slight reduction in activity, suggesting that this site has a certain degree of modification tolerance and that the activity possesses tunable potential.

**Figure 42:**
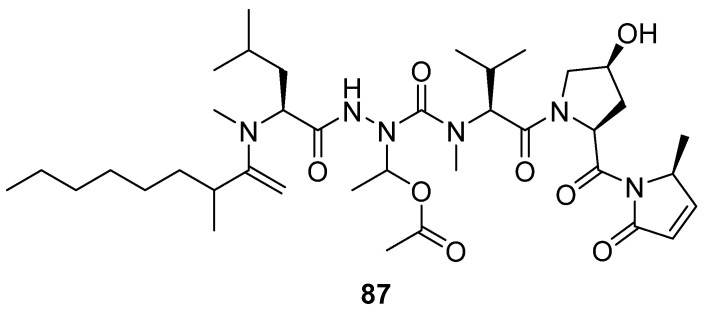
Structure of marine-derived peptide microcolin H (**87**).

In 2023, Wang’s research group synthesized the lipopeptide natural product microcolin H (**87**) derived from the marine organism *Moorea producens* using a total synthesis method (Figure 42) [87]. The activity evaluation results showed that, when assessing its anti-tumor effects using the HGC-27 cell model, the tumor growth inhibition rate (TGI) reached 74.2% in the group treated with 10 mg/kg microcolin H, which was significantly higher than the TGI value of the positive control drug paclitaxel (8 mg/kg). The SAR analysis further revealed that the length of the fatty acid chain is the key structural factor affecting its activity.

In 2024, Wen’s research group conducted in-depth studies [88] based on the marine antibacterial peptide Piscidin-1, which can be isolated from the mast cells of the hybrid striped bass [89]. The activity test results showed that the marine antibacterial peptide Piscidin-1 could inhibit the viability of oral squamous cell carcinoma OC2 and SCC4 cells in a concentration- and time-dependent manner. Within 24 to 72 h, the IC_50_ values of Piscidin-1 for the OC2 and SCC4 cell lines ranged from 10.82 to 13.77 μM and 16.94–19.20 μM respectively, demonstrating a significant anti-proliferative effect.

In 2024, Hao’s research group performed tryptic hydrolysis of phycocyanin, followed by separation, purification and identification of the resulting peptides using ultrafiltration, HPLC, and MS [90]. They identified three predicted peptide segments of phycobilin with anti-tumor activity, namely PCP1 (AGDASVLEDR), PCP2 (ADSLLSGLR), and PCP3 (MFDAFTK). Using CCK-8 assay, anti-tumor activity assays showed that after 24 h of treatment at a concentration of 50 μg/mL, PCP1 reduced the viability of A549 cells to 0.77 μg/mL, H1299 cells to 0.78 μg/mL, and LTEP-a-2 cells to 0.80 μg/mL. PCP2 also showed inhibitory effects at the same concentration, with viability rates of A549, H1299, and LTEP-a-2 cells being 0.76, 0.73, and 0.77 μg/mL, respectively. In contrast, PCP3 at 100 μg/mL reduced the viability of these three cell lines to 0.69, 0.72, and 0.71 μg/mL, respectively. The SAR analysis indicated that acidic amino acid residues could form hydrogen bonds with basic amino acids in EGFR, thereby enhancing the binding ability, while hydrophobic amino acid residues help strengthen hydrophobic interactions with the active pocket of EGFR, further improving the binding stability.

In 2024, Wen’s research group conducted further studies based on the antibacterial peptide Tilapia piscidin 4 (TP4) identified from the Nile tilapia (*Oreochromis niloticus*) by previous researchers [91]. Using MTT assays, anti-tumor activity assays showed that TP4 exhibited IC_50_ values of 6.71 μM against J82 cells and 6.14 μM against T24 cells. In the colony formation experiment, the number of colonies formed by cells treated with TP4 was significantly reduced.

In peptides, amide bonds, hydrophobic amino acid residues (Leu, Phe, Trp), disulfide bridges, and fatty acid chains are most frequent. Major sources are marine cyanobacteria (Hormoscilla, Moorea), sponges (Chondrosia), and mollusks (Arca inflata). Gramicidin A (**83**) shows the highest activity with an IC_50_ of 5.8 nM against P388 leukemia cells; Recifin A (**78**) selectively inhibits TDP1 (IC_50_ = 190 nM) via an allosteric mechanism; P6 peptide achieves 72.66% tumor inhibition in an HT-29 xenograft model, better than 5-fluorouracil. Ion-channel-forming linear peptides or cyclic peptides targeting protein–protein interactions exhibit the best selectivity. Trends from 2020 to 2024 include shifting from extraction to total synthesis and derivative design (e.g., microcolin H); mechanistic studies on membrane depolarization and ion channels; determination of amino acid configuration by Marfey’s method and MS/MS; and repurposing marine antimicrobial peptides as anticancer agents (piscidin-1, TP4).

### 2.6. Marine-Derived Polysaccharides

Marine-derived polysaccharides are structurally diverse macromolecules from seaweeds, fungi, and microorganisms, composed of monosaccharides with varied linkages, sulfation, and branching. Adapted to extreme marine environments, such as high salinity and high pressure, they possess unique chemical and structural features, making them valuable for natural macromolecular drug development and anticancer agent discovery.

In 2022, M. Saied’s research group isolated and purified a novel sulfated polysaccharide EPSR4 from marine *Bacillus subtilis* AC4 sourced from the Red Sea sediment [92]. Structural characterization revealed that the polysaccharide has a molecular weight of 1.48 × 10^4^ g/mol as determined by gel permeation chromatography, and its monosaccharide composition consists of glucose, rhamnose, and arabinose, with a molar ratio of 5:1:3. The FT-IR spectrum further confirmed it as a sulfated polysaccharide with *β*-glycosidic linkages, exhibiting a sulfation degree of 48%. The activity evaluation showed that, compared with the positive control drug cisplatin, compound EPSR4 exhibited weaker cytotoxic activity against bladder cancer T-24 cells (IC_50_ = 244 μg/mL) but stronger cytotoxic activity against lung cancer A549 cells (IC_50_ = 4.08 μg/mL) and hepatocellular carcinoma Hep-G2 cells (IC_50_ = 1.29 μg/mL). Based on existing research, it is speculated that its anti-tumor activity may be closely related to structural features such as the high degree of sulfation, *β*-glycosidic bond configuration, and high crystallinity.

In 2022, S. Ali’s research group isolated an extracellular polysaccharide component, EPSR3, from *Bacillus cereus* AG3 [93]. Structural characterization revealed that this polysaccharide is a non-sulfated acidic heteropolysaccharide with a uronic acid content of 28.7%. The monosaccharide composition analysis indicated that it was composed of glucose, galacturonic acid, and arabinose in a molar ratio of 2.0:0.8:1.0. Gel permeation chromatography determined its weight-average molecular weight to be 1.66 × 10^4^ g/mol, its number-average molecular weight to be 1.37 × 10^4^ g/mol, and its polydispersity index to be 1.2. Infrared spectroscopy analysis revealed characteristic absorption peaks at wave numbers of 3420.14 cm^−1^ (O-H stretching vibration), 1670.05 cm^−1^ (carboxylic acid C = O vibration), 1126.22 cm^−1^ (sugar ring C-O-C vibration), and 832.13 cm^−1^ (α-configuration glycosidic bond). The anti-tumor activity evaluation indicated that EPSR3 exhibited significant inhibitory activity against bladder cancer T-24 cells, breast cancer MCF-7 cells, and prostate cancer PC-3 cells, with IC_50_ values of 121 μg/mL, 55.7 μg/mL, and 61.4 μg/mL, respectively.

In 2023, Ghareeb’s research group isolated an exopolysaccharide designated, EPSF6, from *Bacillus velezensis* AG6 [94]. The structural analysis results indicated that this compound was a non-sulfated acidic heteropolysaccharide, with a uronic acid content of 43.8%. Its monosaccharide composition included xylose, galactose, and galacturonic acid, with a molar ratio of 2.0:0.5:2.0. Gel permeation chromatography determined its weight-average molecular weight (M_w_) to be 2.7 × 10^4^ g/mol, its number-average molecular weight to be 2.6 × 10^4^ g/mol, and the polydispersity index to be 1.1. Fourier transform infrared spectroscopy showed characteristic absorption peaks at 3443.28 cm^−1^ (O-H stretching vibration), 1647.87 cm^−1^ (carboxylic acid C = O stretching vibration), and 864 cm^−1^ (α-glycosidic bond configuration) in the vicinity, further confirming its polysaccharide structure. Activity studies demonstrated that EPSF6 exhibited broad-spectrum inhibitory activity against various tumor cells, with IC_50_ values for HepG2, A-549, HCT-116, MCF-7, Hep-2, and PC-3 cells being 471.88, 532.81, 1089, 483.54, 1586.22, and 450.45 μg/mL, respectively. Although its activity was lower than that of the positive control cisplatin (the percentages of activity calculated based on the IC_50_ of the positive drug cisplatin were 0.273%, 0.766%, 0.217%, 0.705%, 0.265%, and 0.841%), it still showed potential as a natural anti-tumor lead compound.

In 2024, Li’s research group extracted from brown algae *Fucus vesiculosus* and purified it using Q-Sepharose fast-flow ion exchange chromatography, obtaining a new type of brown alginate, MF4 [95]. This substance was identified as a sulfated polysaccharide with an average molecular weight of 67.7 kDa and a sulfate content of 21.5%. The monosaccharide composition included fucosyl, xylose, galactose, glucose, and mannose. IR analysis showed characteristic absorption peaks at 1256.74 cm^−1^, 846.81 cm^−1^, and 1027.92 cm^−1^. Activity evaluation results indicated that in the LLC tumor-bearing mouse model, the in vivo tumor inhibition rate of MF4 was 39.50%, while that of positive control carboplatin-treated group was 64.13%.

### 2.7. Marine-Derived Macrolides

Marine-derived macrolides are characterized by a large polycyclic core scaffold, often featuring complex structural attributes such as highly oxidized modifications, fused-ring systems, and multi-substituted side chains. They exhibit rich stereoscopic chemical diversity and unique marine biosynthetic pathways. Compared to those derived from land sources, these compounds demonstrate greater structural novelty and molecular diversity. As a result, they hold significant importance in the field of natural product chemistry and drug development, continuing to serve as a valuable source of novel lead structures for anticancer drug development.

**Figure 43:**
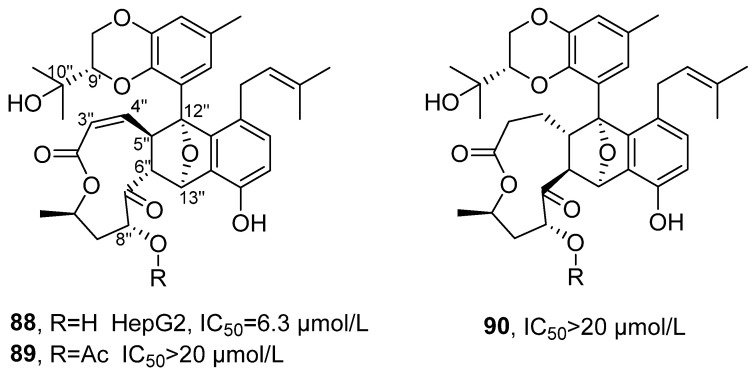
Structures of marine-derived macrolides Lithocarpins E-G (**88–90**).

In 2021, Zhang’s research group isolated three new lithocarpin analogs from the deep-sea fungus *Phomopsis lithocarpus* FS508, and named them Lithocarpins E-G (**88–90**) (Figure 43) [96]. These compounds belong to the rare class of highly oxidized tenellone–macrolide heterodimers in nature, featuring a unique 9,14-epoxy naphtho [2,3-*b*]oxacycloundecin-3(2H)-one skeleton. Taking Lithocarpins E as an example for structural confirmation: its molecular formula was determined to be C_35_H_40_O_9_ with an unsaturation of 16; the presence of a tetrasubstituted benzene ring and an oxygenated methine group was deduced from the ^1^H NMR data. Based on the key HMBC correlation signals, the tenellone unit was confirmed to be connected to the macrocyclic lactone skeleton. Through the key spatial interaction of NOESY, the partial relative configuration was determined, such as H-6″, H-8″ and H-13″ being located on the same side of the macrocyclic lactone ring; the ECD spectrum matched the experimental spectrum, thus determining the absolute configuration of compound 1 as 9′*S*, 5″*R*, 6″*R*, 8″*R*, 10″*R*, 12″*R*, 13″*S*. The in vitro activity test results showed that Lithocarpin E exhibited the strongest activity, with an IC_50_ value of 6.3 μM for HepG2 cells, which was comparable to that of the positive drug cisplatin (2.4 μM). In contrast, Lithocarpins F and G showed significantly reduced activity, with the IC_50_ values for various cell lines being greater than 20 μM. By comparing the activities of Lithocarpins E-G, the SAR revealed that the hydroxyl group at C-8″ and the double bond between C-3″/C-4″ were crucial for activity. Lithocarpins F, which was the acetylated product of the hydroxyl group at C-8″ of 1, displayed decreased activity; Lithocarpins G, the double bond reduction product, showed further diminished activity, indicating that both functional groups were essential structural elements for maintaining high activity.

**Figure 44:**
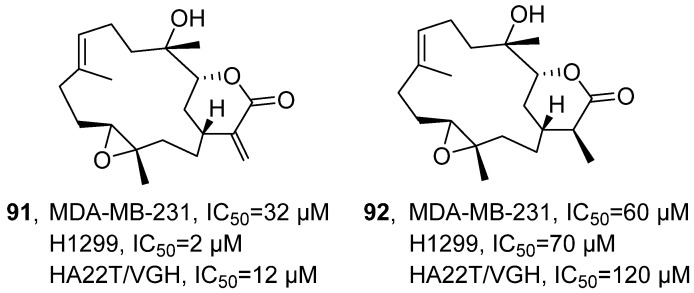
Structures of marine-derived macrolides Sinularin (**91**) and Dihydrosinularin (**92**).

In 2021, Chang’s research group isolated Sinularin (**91**) and Dihydrosinularin (**92**) from the ethyl acetate extracts of the soft corals *Sinularia manaarensis* and *S. flexibilis* (Figure 44) [97]. Both compounds belong to the macrolide class, and their structures were elucidated using NMR spectroscopy. The activity evaluation results showed that the IC_50_ values of Sinularin in MDA-MB-231, H1299 and HA22T/VGH cells were 32 µM, 2 µM and 12 µM respectively; while the IC_50_ values of Dihydrosinularin were 60 µM, 70 µM and 120 µM respectively. The SAR analysis indicated that the differences in antioxidant activity between Sinularin and Dihydrosinularin might be attributed to the presence or absence of conjugated double bonds in their molecular structures.

**Figure 45:**
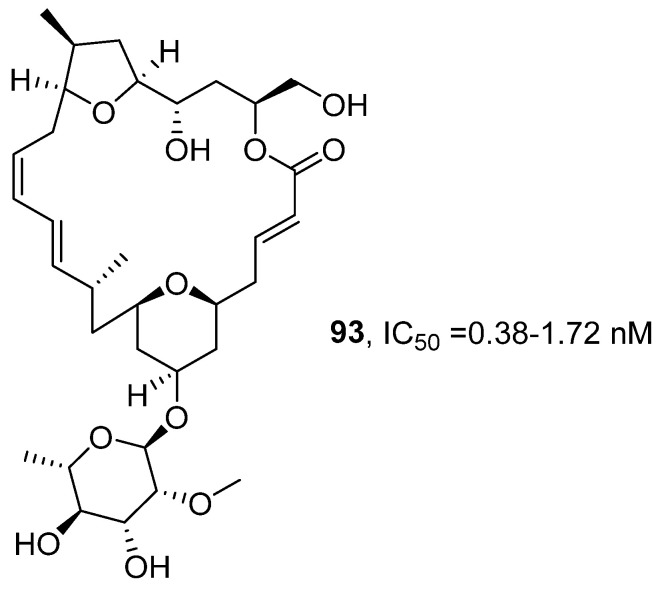
Structures of marine-derived macrolide Mandelalide A (**93**).

In 2022, E. Ishmael’s research group, based on previous work [98], systematically evaluated the anti-tumor activity of the polyketide macrolide natural product Mandelalides (A-L) series compounds (Figure 45) [99]. The study obtained Mandelalide A (**93**) via total synthesis and confirmed its structure by comparing NMR and LC-MS data with those reported in the literature. The activity evaluation results showed that the 48 h EC_50_ value of **93** for wild-type mouse embryonic fibroblast cells (MEF) was 37.1 nM, while its sensitivity to AMPKα-deficient MEF was significantly enhanced (EC_50_ = 13.5 nM). In non-small cell lung cancer cells, regardless of the LKB1 expression status (such as H292, PC-9 being LKB1 positive; H460, 11–18 being LKB1 deficient), the GI_50_ values ranged between 2.0 and 4.7 nM. After extended exposure, **93** showed complete cytotoxicity to all tested glioblastoma cells, with IC_50_ values ranging from 0.38 to 1.72 nM. The SAR analysis further revealed that the type A (featuring a simple lactone linkage) and type B (containing butenolide structure) macrolides could inhibit ATP synthase and activate AMPK, thus showing significant anti-tumor activity. In contrast, Type C macrocycles, which contain a 23-hydroxybutyrolactone structure, had no effect on mitochondrial function and exhibited relatively weak activity. Moreover, **93** displayed stronger cytotoxicity and AMPK activation ability, while the activity of non-glycosylated type C was significantly reduced, indicating that glycosylation is a key structural element for maintaining the strong anti-tumor activity in this class of compounds.

**Figure 46:**
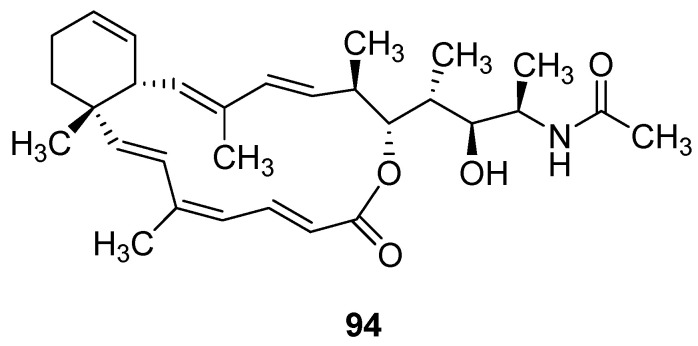
Structure of marine-derived macrolide ZJ-101 (**94**).

In 2023, Liu’s research group further explored the activities of macrocyclic laccoid MNP Superstolide A, based on its analog ZJ-101 (**94**) (Figure 46) [100]. The activity evaluation results showed that **94** exhibited a typical cell growth inhibition effect, with the growth rate inhibition index (GR value) always greater than 0. After 72 h treatment in MDA-MB-231 cells, the GR value reached 0.5 at the highest dose. The EC_50_ measured by the GR method was 29–96 nM. Using triptolide and flavopiridol as cytotoxic control compounds, both of which showed GR values below 0, further supported that **94** primarily functions through growth inhibition. Additionally, **94** showed great activity in 3D tumor sphere models: the EC_50_ for inhibiting tumor sphere formation was approximately 1 nM, while the EC_50_ for disrupting preformed tumor spheroids was about 5 nM, indicating its mechanism of action may be closely related to interfering with the cell adhesion process. In terms of the SAR, as a simplified analog of Superstolide A, **94** not only successfully retained the core biological activity of the parent compound but even enhanced it in some respects, thereby verifying the effectiveness of the structural simplification strategy.

In macrolides, macrocyclic lactone ring, glycosyl groups (especially 2,3-O-dimethylglucose), conjugated dienes, and hydroxyl groups are most frequent. The most active macrolides come from deep-sea fungi (*Phomopsis lithocarpus*), soft corals (*Sinularia, Sarcophyton*), and sponge-associated microbes. Mandelalide A (**93**) exhibits a GI_50_ as low as 2.0–4.7 nM against non-small cell lung cancer cells, among the highest in the literature; Superstolide analog (**94**) shows an EC_50_ of 1 nM for inhibiting 3D tumor sphere formation. Glycosylated macrolides with rigid macrocyclic scaffolds and conjugated enone structures display the best activity and selectivity. Trends from 2020 to 2024 include that total synthesis to overcome supply issues; glycosylation identified as critical for activity (deglycosylation drastically reduces potency); use of GR values to distinguish cytostasis from cytotoxicity; discovery of new mechanisms targeting ATP synthase and the AMPK pathway; and design of simplified analogs (e.g., ZJ-101 (**94**)) that retain core activity while improving synthetic accessibility.

## 3. Skeleton Analysis

Based on the statistics and analysis of literature from 2020 to 2024, it has been found that marine-derived anti-tumor active molecules cover seven major structural classes: terpenoids, alkaloids, sterols, polyketides, peptides, polysaccharides, and macrolides, exhibiting significant scaffold diversity. Different structural types of MNPs show distinct specificity in their anti-tumor activity profiles. Compounds represented by alkaloids demonstrate remarkable cytotoxicity, with their activity often reaching the nanomolar level (e.g., meleagrin has an IC_50_ of 0.03 μM against DU-145 cells). Their SARs clearly point to the nitrogen-containing heterocycle as the core pharmacophore. Macrolides also exhibit significant anti-tumor activity (e.g., mandelalide A shows a GI_50_ of 2.0–4.7 nM), which is highly dependent on glycosylation modifications and the spatial conformation of the macrocyclic scaffold. The activity profile of terpenoids shows significant structural dependency, where specific oxidation patterns and ring system modifications (such as D-ring opening of spongiane diterpenoids) can elevate their activity to the sub-micromolar level. Polyketides achieve selective cytotoxicity through precise stereochemical regulation, with their activity showing a high correlation with chiral center configurations and functional group positioning. Notably, although peptide/protein natural products generally exhibit only micromolar-level activity in conventional cytotoxicity assays, they can transcend the limitations of traditional small molecules by specifically recognizing protein–protein interaction interfaces (e.g., Recifin A shows an IC_50_ of 190 nM against TDP1). In contrast, polysaccharides primarily exert anti-tumor effects by modulating systemic biological processes such as the immune microenvironment and angiogenesis, with their potency closely linked to the degree of sulfation and saccharide chain conformation.

**Figure 47:**
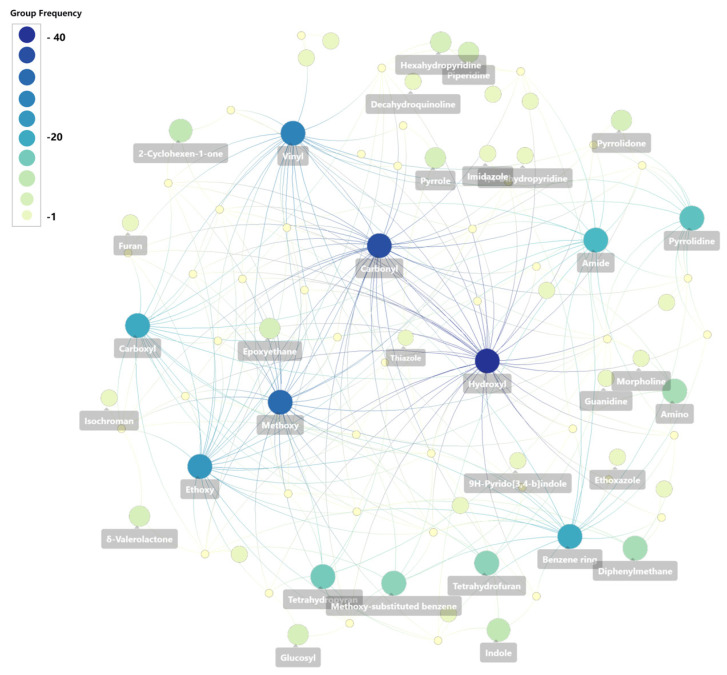
Neo4j knowledge graph of 94 anti-tumor MNPs (2020–2024).

To gain a deeper understanding of the structural features of marine-derived anti-tumor active molecules, this study employed Neo4j knowledge graph technology to systematically analyze the 94 molecules included in this review. Inclusion criteria of 94 MNPs were: (i) basic structural identification information; (ii) reported in vitro anti-tumor activity; and (iii) clear marine origin. Compounds that lacked essential structural data or reported no anti-tumor activity were excluded. Each selected MNP was represented as a node with the label Compound and a secondary label indicating its chemical class (e.g., Terpenoid, Alkaloid). Additionally, each functional group/structural feature (hydroxyl, ketone, methoxy, ethoxy, carboxyl, amide, pyrrolidine, indole, benzene ring, tetrahydropyran, tetrahydrofuran, ester, bromine, thioether) was also represented as a node. The MNP node is connected to the corresponding functional group/structural feature node via edges, indicating the presence or absence of the respective features in the MNP. Data were imported from CSV files using the LOAD CSV command with MERGE statements to avoid duplicate nodes. Constraints were created on Compound.name and FunctionalGroup.name to accelerate queries. The analysis was performed using Cypher queries, e.g., MATCH (c:Compound)-[:HAS_GROUP]->(f:FunctionalGroup) RETURN f.name, count(c), to count the frequency of each functional group across the dataset. The resulting distribution, visualized in Figure 47, revealed that these functional groups are primarily composed of carbon, oxygen, and nitrogen. Among them, oxygen-containing functional groups dominate. The five most frequently occurring groups are hydroxyl (39 occurrences), ketone (35 occurrences), methoxy (27 occurrences), ethoxy (24 occurrences), and carboxyl (20 occurrences). These groups collectively form the core hydrogen-bonding network of the molecules, directly impacting their crucial interactions with biological targets. The predominance of oxygen-containing functional groups further indicates that the scaffold distribution of marine anti-tumor compounds is not monomodal but rather presents a complex network characterized by a high oxidation state. Nitrogen-containing functional groups appear less frequently, with amide (16 occurrences), pyrrolidine (6 occurrences), and indole (3 occurrences) being relatively common. It is noteworthy that among the active molecules reported in the past five years, alkaloids have exhibited particularly significant anti-tumor activity. For example, the alkaloid Meleagrin (**25**) shows an IC_50_ of 0.03 μM against prostate cancer DU-145 cells, which is significantly superior to the positive control drug doxorubicin (IC_50_ = 0.34 μM). This demonstrates that the introduction of nitrogen atoms significantly influences the anti-tumor activity of marine natural molecules. Furthermore, elements such as bromine, sulfur, and chlorine are present in some molecules, reflecting the structural characteristics of MNPs. The high frequency of benzene rings (19 occurrences) indicates that aromatic rings are key modules in marine bioactive scaffolds, conferring structural rigidity, π-π stacking ability, and specific three-dimensional conformations. Heterocyclic structures such as tetrahydropyran (8 occurrences), tetrahydrofuran (6 occurrences), indole (3 occurrences), and pyrrolidine (6 occurrences) appear relatively frequently, further indicating that oxygen/nitrogen-containing heterocycles are important components of marine bioactive scaffolds (Figure 47). The common presence of tetrahydropyran and tetrahydrofuran suggests that polyethers and macrolides may possess certain activity advantages. These oxygen-containing heterocycles often act as rigid scaffolds, maintaining the spatial conformation of the molecule and allowing it to fit precisely into receptor binding pockets. The high frequency of nitrogen-containing scaffolds such as amides, pyrrolidines, and indoles suggests that marine alkaloids and peptides may have certain advantages in activity, as these scaffolds are often associated with potent cytotoxicity. Regarding connectivity, amide bonds, ester bonds, and aliphatic chains are the main linking units, combining structural modules into linear, fused-ring, or macrocyclic forms. Among these, fused-ring structures demonstrate the best biological activity within the existing data.

## 4. Target Prediction

**Figure 48:**
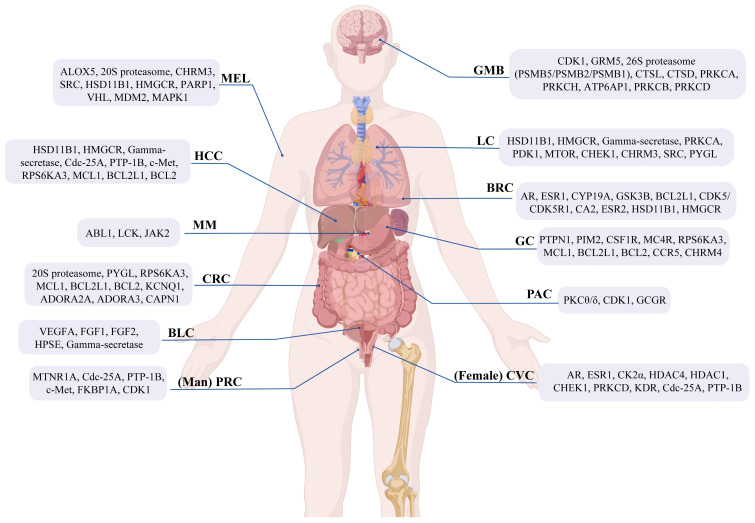
Predicted targets of anti-tumor MNPs (2020–2024).

Based on the statistical analysis of literature from 2020 to 2024, research on the anticancer activity of MNPs has been highly concentrated on three major prevalent cancer types: breast cancer (mentioned 14 times), prostate cancer (5 times), and lung cancer (12 times). This focus directly correlates with their global disease burden and urgent clinical need. It is noteworthy that some compounds have demonstrated remarkable potency. For instance, Meleagrin (**25**) exhibits an IC_50_ as low as 0.03 μM against prostate cancer DU-145 cells, and Sphaerococcenol A shows an IC_50_ of only 0.70 μM against colon cancer stem cell HT29 spheroids. These nanomolar-level activities signify that these compounds possess key characteristics of promising lead candidates. However, recent research on marine-derived anti-tumor active molecules has primarily focused on activity screening, while exploration of their potential molecular targets remains relatively scarce. To address this, we selected MNPs with well-defined chemical structures and reported anti-tumor activity between 2020 and 2024. Their structures were submitted to the SwissTargetPrediction platform, which generated binding scores against a wide range of potential targets. For each molecule, the top five predicted targets (i.e., those with the highest scores) were systematically collected and subjected to in-depth analysis. The results revealed that MNPs reported from 2020 to 2024 exhibit the following target profile characteristics for different tumor types (Figure 48).

The predicted target combinations of MNPs for various cancers are primarily enriched in core biological processes. For glioblastoma, MNPs can interfere with neuronal excitability regulation, protein degradation pathways, and kinase-signaling networks by targeting GRM5, 26*S* proteasome subunits (PSMB5, PSMB2, PSMB1), and PKC family members (PRKCA, PRKCH, PRKCB, PRKCD), thereby affecting tumor metabolism and inducing cell death. In melanoma, MNPs target ALOX5, the 20*S* proteasome, VHL, PARP1, MDM2, RORC, MAPK1, PRKCD, etc., to modulate inflammation and oxidative stress, protein homeostasis, genomic stability, and signaling pathways, ultimately inhibiting tumor survival. For lung cancer, MNPs cover targets such as EGFR, c-Met, VEGFA, FGF1, FGF2, HSD11B1, HMGCR, PSEN1, PSEN2, CCR5, and CCR1, intervening in proliferation, metabolic reprogramming, angiogenesis, and the immune microenvironment to promote cancer cell elimination.

In multiple myeloma, MNPs modulate non-receptor tyrosine kinase signaling networks through ABL1, LCK, and JAK2, disrupting proliferation, immune microenvironment communication, and cytokine signaling. For breast cancer, MNPs target AR, ESR1, ESR2, CYP19A1, HSD11B1, HMGCR, CA2, GSK3B, CDK5/CDK5R1, BCL2L1, etc., affecting hormone signaling, metabolic reprogramming, cell fate decisions, and resistance to cell death. In liver cancer, MNPs act on HSD11B1, HMGCR, CYP19A1, MCL1, BCL2L1, BCL2, XIAP, VEGFA, FGF1, FGF2, HPSE, CFD, F10, proteasome components, PRKCA, MET, SYK, etc., disturbing metabolism, apoptotic resistance, angiogenesis, and protein homeostasis to suppress tumor progression.

For other cancers, MNPs similarly focus on key processes: in pancreatic cancer, they target PKCθ/δ, CDK1, and GCGR to intervene in signaling nodes and the cell cycle; in gastric cancer, they act on CSF1R, MC4R, CHRM2, CHRM4, CHRM5, PIM2, and PTPN1 to affect microenvironment communication and signal transduction; in colorectal cancer, they regulate PYGL, STAT3, ADORA2A, ADORA3, ITGA4, KCNQ1, and CAPN1 to interfere with metabolic adaptation and the immune microenvironment; in bladder cancer, they concentrate on VEGFA, FGF1, FGF2, HPSE, and Gamma-secretase to inhibit angiogenesis and extracellular matrix remodeling; in cervical cancer, they target AR, ESR1, CK2α, KDR, PDE4B, and LCK for coordinated intervention in hormone, kinase, and immune signaling; and in prostate cancer, they affect MTNR1A, Cdc-25A, CDK1, PTP-1B, c-Met, and FKBP1A, covering neuroendocrine, cell cycle, and growth factor signaling networks. Through this multi-layered and coordinated intervention, MNPs ultimately effectively inhibit the growth and induce the death of various cancer cells.

## 5. Conclusions and Perspectives

Based on the statistical analysis of literature from 2020 to 2024, MNPs with anti-tumor activity are classified into seven major categories: terpenoids, alkaloids, sterols, polyketides, peptides/proteins, polysaccharides, and macrolides. This classification fully reflects the remarkable chemical diversity of marine-derived metabolites. Compounds from different structural classes exhibit distinct specificity in their anti-tumor activities. Anticancer research has been heavily concentrated on three highly prevalent cancer types: breast cancer (mentioned 14 times), prostate cancer (5 times), and lung cancer (12 times). This focus directly corresponds to their significant global disease burden and urgent clinical needs, not only revealing current research priorities but also highlighting the vast potential of MNPs in addressing major public health challenges.

Notably, certain compounds demonstrate impressive potency. For instance, Meleagrin (**25**) exhibits an IC_50_ as low as 0.03 μM against prostate cancer DU-145 cells, compound **7** shows a GI_50_ of 4.21 μM against triple-negative breast cancer MDA-MB-231 cells, and Sphaerococcenol A has an IC_50_ of only 0.70 μM against colon cancer stem cell HT29 spheroids. These micromolar and even nanomolar-level activities signify that they possess key characteristics of promising lead compounds. Concurrently, the broad-spectrum activity observed for some MNPs (e.g., Sphaerococcenol A and various polysaccharides) suggests they may exert anti-tumor effects by targeting multiple points or unique pathways. This offers novel perspectives for overcoming the drug resistance commonly associated with traditional chemotherapy.

In addition, this review employed Neo4j knowledge graph technology to statistically analyze the chemical scaffolds of 94 marine anti-tumor molecules, systematically revealing the structural commonalities of MNPs. The pharmacophoric scaffold of these carbon-oxygen-nitrogen-based molecules integrates both nitrogen-containing heterocycles and oxygen functional groups. While oxygen groups, hydroxyl (39), ketone (35), methoxy (27), ethoxy (24), carboxyl (20), dominate in frequency and form an extensive hydrogen-bond network, reflecting high oxidation, nitrogen-containing motifs like amide (16), pyrrolidine (6), and indole (3), are equally critical, jointly determining bioactivity. The benzene ring (19) serves as a key rigid module. Recurrent heterocycles such as tetrahydropyran (8), tetrahydrofuran (6), indole, and pyrrolidine further highlight their structural importance. Connected mainly by amide/ester bonds and aliphatic chains, these modules assemble into diverse architectures, offering valuable insights for marine natural product-based anticancer drug design. And target prediction analysis of representative molecules using the SwissTargetPrediction platform revealed that marine anti-tumor active molecules may act on multiple key signaling nodes, including kinases, proteasomes, nuclear receptors, cell cycle regulatory proteins, epigenetic modifiers, and immunomodulatory targets. This multi-target, network-based action profile not only predicts the potential mechanism behind broad-spectrum activity of MNPs, but also provides a theoretical foundation for designing novel mechanism-based combination therapy strategies.

Despite the immense potential of MNPs in anticancer drug discovery, significant challenges remain. Current bioactivity screening is heavily biased toward conventional in vitro cytotoxicity assays on a narrow panel of cancer cell lines. Such reductionist models fail to capture the complexity of the tumor microenvironment and frequently overlook compounds with non-cytotoxic but therapeutically relevant activities, such as immunomodulation or cancer stem cell inhibition. Furthermore, the translation of exciting nanomolar activities into druggable leads is hindered by insufficient mechanism-of-action validation. To bridge these gaps, future research should focus on the following directions: (1) Expanding marine drug sources and achieving sustainable production by integrating genomics, metabolomics, and synthetic biology technologies, as well as developing innovative synthetic routes to secure reliable access to complex scaffolds; (2) Conducting systematic in vivo pharmacodynamic, pharmacokinetic, and safety evaluations to advance lead compounds toward preclinical candidate drugs; (3) Elucidating molecular action mechanisms of MNPs, with particular attention to their unique roles in modulating the tumor microenvironment, activating immunity, and inhibiting cancer stem cells, and integrating chemical proteomics and genetic target deconvolution early in the discovery workflow; and (4) Adopting a systems-level approach that leverages artificial intelligence for bioactivity and ADMET prediction, constructs comprehensive MNP-focused databases, and fosters multidisciplinary collaboration to streamline the hit-to-lead process.

## Data Availability

The raw data supporting the conclusions of this article will be made available by the authors on request.
